# Multicenter validation of secondary hemophagocytic lymphohistiocytosis diagnostic criteria

**DOI:** 10.1111/joim.20065

**Published:** 2025-01-27

**Authors:** Gunnar Lachmann, Patrick Heeren, Friederike S. Schuster, Peter Nyvlt, Claudia Spies, Insa Feinkohl, Thomas Schenk, Wafa Ammouri, France Debaugnies, Lionel Galicier, Yuan Jia, Nikhil Meena, Carole Nagant, Olaf Neth, Stefan Nierkens, Juan San Martin, Hao Wei (Linda) Sun, Yini Wang, Zhao Wang, Jae‐Ho Yoon, Frank M. Brunkhorst, Paul La Rosée, Gritta Janka, Cornelia Lachmann

**Affiliations:** ^1^ Department of Anesthesiology and Intensive Care Medicine (CCM, CVK) Charité—Universitätsmedizin Berlin, Corporate Member of Freie Universität Berlin and Humboldt‐Universität zu Berlin Berlin Germany; ^2^ Berlin Institute of Health at Charité—Universitätsmedizin Berlin Berlin Germany; ^3^ Institute of Medical Informatics Charité—Universitätsmedizin Berlin Berlin Germany; ^4^ Medical Biometry and Epidemiology Group Witten/Herdecke University Witten Germany; ^5^ Max‐Delbrueck‐Center for Molecular Medicine in the Helmholtz Association (MDC), Molecular Epidemiology Research Group Berlin Germany; ^6^ Department of Hematology and Oncology Universitätsklinikum Jena Jena Germany; ^7^ Internal Medicine Department IBN SINA Hospital Faculty of Medicine and Pharmacy Mohammed V University Rabat Morocco; ^8^ Laboratory of Translational Research Centre Hospitalier Universitaire Brugmann, Université libre de Bruxelles Brussels Belgium; ^9^ Department of Laboratory Medicine Centre Hospitalier de Luxembourg Luxembourg Luxembourg; ^10^ Internal Medicine, Hôpital La Timone, APHM Marseille France; ^11^ Department of Rheumatology and Immunology Peking University People's Hospital Beijing China; ^12^ Department of Internal Medicine Pulmonary and Critical Care Medicine University of Arkansas for Medical Sciences Little Rock Arkansas USA; ^13^ Immunology Department LHUB‐ULB, Université libre de Bruxelles Brussels Belgium; ^14^ Paediatric Infectious Diseases Rheumatology and Immunology Unit Hospital Universitario Virgen del Rocío, Instituto de Biomedicina de Sevilla, IBiS/Universidad de Sevilla/CSIC Seville Spain; ^15^ Center for Translational Immunology (CTI), UMC Utrecht Utrecht The Netherlands; ^16^ Princess Máxima Center for Pediatric Oncology Utrecht The Netherlands; ^17^ Hospital Universitario de Fuenlabrada Madrid Spain; ^18^ Biomedical Research Center Network in Infectious Diseases (CIBERINFEC) Madrid Spain; ^19^ Department of Medicine Division of Hematology (DoM) University of Alberta Edmonton Canada; ^20^ Department of General Medicine Beijing Friendship Hospital, Capital Medical University Beijing China; ^21^ Department of Hematology Beijing Anzhen Hospital, Capital Medical University Beijing Beijing China; ^22^ Hematology Department Beijing Friendship Hospital, Capital Medical University Beijing Beijing China; ^23^ Department of Hematology Catholic Hematology Hospital and Leukemia Research Institute, Seoul St. Mary's Hospital, College of Medicine, The Catholic University of Korea Seoul South Korea; ^24^ Department of Anesthesiology and Intensive Care Medicine Universitätsklinikum Jena Jena Germany; ^25^ Department of Internal Medicine Schwarzwald‐Baar‐Klinikum Villingen‐Schwenningen Germany; ^26^ Clinic of Pediatric Hematology and Oncology University Medical Center Eppendorf Hamburg Germany

**Keywords:** hemophagocytic lymphohistiocytosis (HLH), hemophagocytic syndrome (HPS), HLH‐2004 criteria, HScore, macrophage activation syndrome (MAS), validation

## Abstract

**Background:**

Five fulfilled hemophagocytic lymphohistiocytosis (HLH)‐2004 criteria, and the HScore are widely used and recommended by international expert consensus to diagnose secondary HLH. Both diagnostic scores have never been validated in heterogeneous patient cohorts of secondary HLH patients. We aimed to systematically optimize and validate diagnostic criteria of secondary HLH using a multicenter approach.

**Methods:**

We developed optimized criteria in our cohort of critically ill patients as a first step. We next validated these new criteria together with the original and modified HLH‐2004 criteria as well as the HScore using original data of 13 published cohorts, which were identified by a systematic literature search.

**Results:**

The best performing HLH diagnostic criteria sets over all 13 validation cohorts were the original HLH‐2004 criteria with a decreased cut‐off (cut‐off 4, mean sensitivity 86.5%, mean specificity 86.1%), followed by the revised HLH‐2004 criteria (natural killer cell activity removed; cut‐off 4, mean sensitivity 83.8%, mean specificity 87.8%) and the HScore (cut‐off 169, mean sensitivity 82.4%, mean specificity 87.6%). Our newly developed HLH diagnostic criteria showed inferior performance. Ferritin ≥500 µg/L had 94.0% mean sensitivity over all cohorts.

**Conclusions:**

In this first multicenter validation study, four fulfilled HLH‐2004 criteria and an HScore of 169 were suitable to diagnose secondary HLH, which will lead to rapid diagnosis and improved patient outcomes. Ferritin proved as a reliable HLH screening marker. Our results should be taken into account in clinical recommendations and in designing new studies.

## Graphical abstract

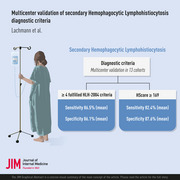

**Abbreviations**
aHLH‐2004: adjusted HLH‐2004 criteria
ASAT: aspartate aminotransferase
HLH: hemophagocytic lymphohistiocytosis
HPS: hemophagocytic syndrome
ICU: intensive care unit
MAS: macrophage activation syndrome
NK: natural killer
OHI: optimized HLH inflammatory
oHLH‐2004: optimized HLH‐2004 criteria
pHLH: primary hemophagocytic lymphohistiocytosis
revHLH‐2004: revised HLH‐2004 criteria
ROC: receiver operating characteristics
shHLH‐2004: shortened HLH‐2004 criteria
sHLH: secondary hemophagocytic lymphohistiocytosis
sIL‐2R: soluble interleukin‐2 receptor

## Introduction

Secondary hemophagocytic lymphohistiocytosis (HLH), also known as macrophage activation syndrome (MAS) or hemophagocytic syndrome (HPS), is a rare though potentially life‐threatening immune disorder characterized by uncontrolled immune activation, inflammation, and organ damage [1] and is associated with high mortality rates of up to 60% [2]. Due to the nonspecific nature of its symptoms and the lack of a single diagnostic test, diagnosis of HLH is challenging as rather complex parameters are applied. This contributes to a high rate of undiagnosed HLH cases, which is reported to be 78% in adult critically ill patients [1].

The HLH‐2004 criteria, developed by the Histiocyte Society, are widely used and recommended by international expert consensus to diagnose secondary HLH [3]. They consist of a combination of clinical and laboratory findings, of which 5 out of 8 or a molecular diagnosis consistent with HLH needs to be fulfilled in order to confirm the diagnosis [3, 4]. They were recently revised for the diagnosis of primary HLH (pHLH): Natural killer (NK) cell activity was removed, and consequently, 5 out of 7 criteria need to be fulfilled [5]. Originally, these criteria, established by the Histiocyte Society HLH‐2004 study group, were intended to be used in a clinical research setting in children [6]. The cut‐offs initially relied on expert consensus but have now been confirmed in a data‐driven study based on a large number of cases and controls [5]. However, no validation of the HLH‐2004 criteria has been performed in secondary HLH patients yet. In addition, the HLH‐2004 criteria's applicability is limited by the fact that some parameters included in the criteria are not routinely available in all hospitals (e.g., NK cell activity, soluble interleukin‐2 receptor [sIL‐2R]), which can contribute to delayed diagnosis. The eponymous hemophagocytosis requires an invasive procedure and a high level of expertise [7]. It has also been argued that the HLH‐2004 criteria lack sensitivity, particularly in early stage HLH, as some criteria will occur—if at all—only during the disease course (e.g., hemophagocytosis) [8].

The HScore has been proposed as an alternative approach to diagnose secondary HLH in adults. Fardet et al. [9] developed a diagnostic scoring system by which the probability of HLH is calculated based on several clinical and laboratory parameters. However, when interpreting a patient's HScore, it needs to be considered that this value has to be seen in relation to the original cohort in Fardet's study. Of note, the majority of patients included in the original study population had infections or malignancies as underlying diseases, limiting the applicability of the HScore in patients with autoimmune/autoinflammatory‐triggered HLH [9]. Therefore, it has already been suggested to modify the HScore according to the respective population of interest [10]. In a recent retrospective multicenter study, Zoref‐Lorenz et al. [11] presented a new tool, the optimized HLH inflammatory (OHI) index, for early HLH detection using the combination of sIL‐2R and ferritin. The authors increased the cut‐offs of both parameters and thereby achieved sensitivity and specificity of 84% and 81%, respectively, to accurately identify HLH in patients with hematologic malignancies [11].

Previous studies sought to analyze the performance of both HLH‐2004 criteria and HScore in different retrospective cohorts with overall satisfying results [10, 12–15]. Sensitivity and specificity for five fulfilled HLH‐2004 criteria to diagnose HLH ranged from 70% to 91% and 93% to 99%, respectively [10, 12–15]. As for the HScore, the authors mostly reported thresholds lower than the suggested score of 169 by the original publication [9, 10, 15]. Of note, all previous investigations were retrospective in nature and contained a considerable risk of bias due to the amount of missing data. Moreover, both the HLH‐2004 criteria and the HScore have never been validated in heterogeneous patient cohorts of secondary HLH patients. Therefore, we aimed to systematically optimize and validate diagnostic criteria of secondary HLH using a multicenter approach.

## Methods

### Inhouse optimization of HLH diagnostic criteria

The basis of our optimizing process (Fig. S1) was the dataset of our previously published study [15, 16] containing 2623 adult critically ill patients, of whom 40 were diagnosed with secondary HLH [17]. Within the study, all clinical variables of non‐HLH patients were recorded on the day of maximum ferritin or extended to a defined time range if not assessed on the respective day. For HLH patients, the respective most pathological clinical values during the entire intensive care unit (ICU) stay were used. We trained a model on this dataset to systematically optimize diagnostic criteria. First, we optimized the specific cut‐offs of each single criterion of the original HLH‐2004 criteria (Table S1), resulting in optimized HLH‐2004 criteria (oHLH‐2004) (oHLH‐2004). Next, we created new sets of HLH diagnostic criteria (each numbered Iteration X), for which we modified HLH‐2004 criteria based on clinical experience and availability (Table 1). For each iteration, optimal cut‐offs were determined for every single criterion, with corresponding optimal numbers of fulfilled HLH diagnostic criteria. The best performing iterations to diagnose HLH within our dataset were selected for validation.

**Table 1:** Our newly developed hemophagocytic lymphohistiocytosis (HLH) diagnostic criteria sets, modified from HLH‐2004 criteria.

	Ferritin	Fever	Splenomegaly	Hepatomegaly	Cytopenias in ≥2 lines	Hypertriglyceridemia and/or hypofibrinogenemia	Hemophagocytosis	Reduced NK cell activity	sIL‐2R	ASAT
Iteration 1	+	+	+	−	+++	+	+	+	+	−
Iteration 2	+	+	+	−	+	+	−	−	+	−
Iteration 3	+	+	+	−	+	+	−	−	−	−
Iteration 4	+	+	+	−	+++	+	−	−	−	−
Iteration 5	+	+	+	−	+++	++	−	−	−	−
Iteration 6	+	+	+	−	+++	++	−	−	−	+
Iteration 7	+	+	+	+	+++	++	−	−	−	−
Iteration 8	+	+	+	+	+++	++	−	−	−	+
Iteration 9	+	+	+	+	+	+	−	−	−	−
Iteration 10	+	+	+	−	+	+	−	−	−	+
Iteration 11	+	+	+	−	+++	+	−	−	+	−
Iteration 12	+	+	+	−	+++	++	−	−	+	−
Iteration 13	+	+	+	−	+++	++	−	−	+	+

*Note*: + included criterion; − excluded criterion; ++ included criterion but each as a separate criterion; +++ included criterion but each cell line as a separate criterion.

Abbreviations: ASAT, aspartate aminotransferase; NK, natural killer; sIL‐2R, soluble interleukin‐2 receptor.

### Acquisition of validation cohorts

For the acquisition of suitable validation cohorts, we conducted a systematic literature search of the MEDLINE (PubMed) database. We used the following search term to retrieve broad results: ((“hemophagocytic lymphohistiocytosis”) OR (“haemophagocytic lymphohistiocytosis”) OR (“haemophagocytic syndrome”) OR (“hemophagocytic syndrome”) OR (“macrophage activation syndrome”)) AND ((“study”) OR (“studies”)). To obtain a suitable sample size for validation, all original studies reporting ≥10 HLH patients among ≥100 patients of all ages were included (“validation datasets”). Studies of only HLH patients were not considered. Corresponding authors were contacted and asked to provide variables of interest of their data (diagnosed HLH, hepatomegaly, splenomegaly, known underlying immunosuppression, core body temperature, ferritin, sIL‐2R, triglycerides, fibrinogen, hemoglobin, thrombocytes, leukocytes, hemophagocytosis in bone marrow or spleen or lymph nodes, reduced NK cell activity, and aspartate aminotransferase [ASAT]), of which the most pathological values regarding HLH were requested. As our cohort [15] had a missing data rate of 31.0% of the 15 variables of interest, we excluded all cohorts that exceeded 31.0% of missing data. Patients with pHLH were removed from the original datasets. To ensure scientific quality, we did not request HLH‐2004 criteria and HScore from the original cohorts but recalculated them using the same algorithm for all cohorts. Fever was defined as core body temperature ≥38.3°C [18], and leukopenia was defined as leukocyte count < 1.67/nL [15] for this purpose.

### Validation of original, modified and our newly developed HLH diagnostic criteria sets

For validation, we considered the original HLH‐2004 criteria [4], adjusted HLH‐2004 criteria (aHLH‐2004): cut‐offs adjusted to ferritin ≥3000 µg/L and core body temperature ≥38.2°C [15], oHLH‐2004, revised HLH‐2004 criteria (revHLH‐2004): NK cell activity removed [5], shortened HLH‐2004 criteria (shHLH‐2004): hemophagocytosis and NK cell activity removed, HScore [9] (Table S2), OHI index (positive for sIL‐2R > 3900 U/mL and ferritin > 1000 µg/L) [11], and best performing iterations within our dataset. As ferritin and sIL‐2R are biomarkers of interest in HLH diagnosis, these were also analyzed separately for validation. All HLH diagnostic criteria sets and biomarkers were tested in each single validation cohort, of which the best sets to discriminate between HLH and non‐HLH patients were determined.

As a post hoc analysis, we analyzed the performance of ferritin as HLH screening marker.

### Statistical analysis

Descriptive statistics are shown as median ± quartiles or count with percentage, respectively. Calculation of optimal cut‐off combinations and corresponding optimal numbers of fulfilled diagnostic criteria was done by using an exhaustive grid search method for cut‐off optimization in our dataset to systematically narrow down optimal cut‐offs: First, a cut‐off range for each HLH‐2004 criterion was determined considering the minimum and maximum values of the 40 HLH patients. Each cut‐off range was divided into 10 equal parts, resulting in a set of 10 cut‐offs for each criterion (Table S3). Second, receiver operating characteristics (ROC) analyses with HLH diagnosis as outcome variable and the number of fulfilled HLH‐2004 criteria as test variable were performed for all cut‐off combinations. The highest Youden's indices determined the optimal cut‐off combinations and the corresponding optimal number of fulfilled HLH‐2004 criteria. Third, new cut‐off ranges for each HLH‐2004 criterion were created considering the cut‐off combinations with Youden's index ≥0.97 of the previous step, the distribution of the respective values of HLH and non‐HLH patients, and the clinical meaningfulness. Each new cut‐off range was again divided into 10 equal parts, resulting in a new set of 10 cut‐offs for each criterion (Table S4). Fourth, Step 2 was repeated with optimized cut‐offs from Step 3, resulting in oHLH‐2004. This step was also repeated for all iterations to determine their optimal cut‐off combinations and their corresponding optimal number of fulfilled HLH diagnostic criteria. Best performing iterations (Youden's index ≥0.975) were considered for validation. If one iteration showed more than one optimal cut‐off combination with equal Youden's indices, the combination closest to the original HLH‐2004 criteria was used.

For validation, accuracy, sensitivity, specificity, as well as positive and negative predictive values were determined by ROC analyses with HLH diagnosis as outcome variable. All considered HLH diagnostic criteria sets were tested in each single validation cohort. A Youden's index mean among all validation cohorts was calculated for each set of diagnostic criteria to determine the best performing set. A sign test was used to indicate whether the alternative criteria were significantly inferior compared to the best performing criteria. As a sensitivity analysis, we rerun all validation analyses again for patients who had at least 5 or 6 obtained HLH‐2004 criteria in their dataset, respectively, to reduce the missing data bias (Tables S5 and S6, respectively). Another sensitivity analysis was conducted for cohorts with HLH diagnosis based on expert review (Table S7).

Ferritin was evaluated using ROC analyses with HLH diagnosis as outcome variable. SPSS Statistics, version 26.0 software (IBM Corporation, Armonk, NY, USA), and The R Statistical Software (version 3.6.3) were used for the analysis. For the calculation of prediction performance measures, the caret package (version 6.0–86) was used. A *p*‐value < 0.05 was considered statistically significant.

### Ethics

Ethics approval was obtained from the institutional review board (Ethikkommission der Charité—Universitätsmedizin Berlin, EA1/176/16). The study was registered with www.ClinicalTrials.gov (NCT02854943) on August 1, 2016.

## Results

### Performance of our newly developed HLH diagnostic criteria sets within our dataset

Patient and outcome characteristics of our dataset, including diagnosis findings by expert consensus, were previously described in detail [15, 16, 17]. oHLH‐2004, as well as our newly developed HLH diagnostic criteria sets with best cut‐off combinations and quality criteria within our dataset, are shown in Table S8. Iteration 13, with a cut‐off of 7 fulfilled criteria, showed the best performance in our dataset (sensitivity 100%, specificity 99.3%). The best performing iterations chosen for validation were iterations 1, 2, 5, 6, 8, 11, 12, and 13.

### Acquisition and characteristics of the validation cohorts

**Fig. 1:**
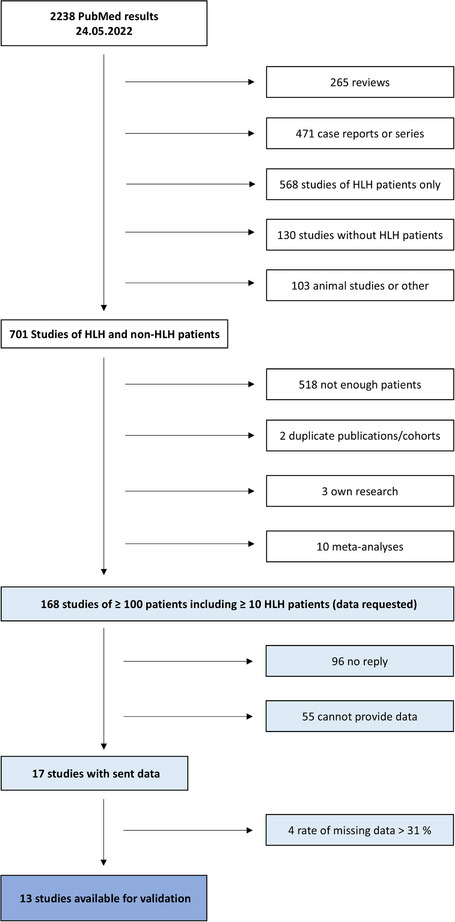
Flowchart of study selection.

We received the requested data from 17 studies, of which 13 were suitable as validation cohorts (Fig. 1). Of these, two cohorts reported ICU patients only (Meena et al. [14] and Debaugnies et al. [19]). Characteristics of the validation cohorts are shown in Table 2. Table S9 describes the requested data and missing data rates of the cohorts in detail.

**Table 2:** Characteristics of the validation cohorts.

			sHLH trigger diseases^a^			HLH patients with Ferritin < 500 µg/L (%)					
	Total patient cohort analyzed	HLH patients	Hematologic malignancy	Infection	Rheumatologic disease		Design	Region	Diagnosis of HLH	Overall missing data rate^b^ (%)	Remarks
Fardet et al. 2016 [9]	266 adults	162 sHLH	57%	25%	3%	1.3	Retrospective, multicenter	France	Expert consensus	14.9	Exclusion of 46 undetermined cases
Debaugnies et al. 2016 [10]	71 children + 68 adults	35 sHLH + 1 pHLH (16 children + 20 adults)	13% for children, 15% for adults	50% for children, 35% for adults	25% for children, 0% for adults	0	Retrospective, multicenter	Belgium	Expert consensus	20.5	Exclusion of 1 pHLH patient, exclusion of 7 undetermined cases
Horrillo et al. 2019 [20]	111 patients with visceral leishmaniasis (≥14 years)	42 sHLH	0%	100%	0%	0	Prospective, monocenter	Spain	HLH‐2004 criteria	15.6	Fever initially as dichotomous variable^c^
Meena et al. 2020 [14]	445 adult ICU patients	10 sHLH	60%	10%	10%	0	Retrospective, monocenter	USA	Expert consensus	30.0	
Debaugnies et al. 2021 [19]	120 adult ICU patients	14 sHLH	29%	43%	0%	0	Prospective, multicenter	Belgium	Expert consensus	17.9	
Lopez Marcos et al. 2021 [21]	127 children with visceral leishmaniasis (≤14 years)	37 sHLH	0%	100%	0%	10.7	Retrospective, multicenter	Spain	HLH‐2004 criteria	29.1	Fever initially as dichotomous variable^c^
Smits et al. 2021 [22]	149 pediatric and adult patients	16 pHLH + 70 sHLH	26%	29%	16%	8.7	Retrospective, multicenter	The Netherlands	HLH‐2004 criteria	29.9	Exclusion of 16 pHLH patients, fever initially as dichotomous variable^c^, sIL‐2R initially in pg/mL^d^
Oh et al. 2021 [23]	101 patients (≥15 years)	60 sHLH	31%	45%	0%	5.0	Prospective, monocenter	South Korea	HLH‐2004 criteria	4.0	Exclusion of 22 unclear MAS cases
Yao H et al. 2021 [24]	168 adults with adult‐onset Still's disease	56 sHLH	0%	0%	100%	0	Retrospective, multicenter	China	Expert consensus	9.6	sIL‐2R initially in pg/mL^d^
Yao S et al. 2021 [25]	348 patients with lymphoma (≥13 years)	104 sHLH	100%	0%	0%	9.7	Prospective, monocenter	China	HLH‐2004 criteria	13.9	sIL‐2R initially in pg/mL^d^
Ammouri et al. 2022 [26]	208 adults with systemic lupus erythematosus	20 sHLH	0%	0%	100%	0	Retrospective, monocenter	Morocco	HLH‐2004 criteria	25.5	Neutrophils instead of leukocytes available^e^
He et al. 2022 [27]	45 children/adolescents + 76 adults with chronic active Epstein–Barr virus infection	73 sHLH (26 children/adolescents + 47 adults)	0%	100%	0%	34.8	Retrospective, monocenter	China	HLH‐2004 criteria	1.6	sIL‐2R initially in pg/mL^d^
Bilston et al. 2022 [12]	897 adults	98 sHLH	34%	31%	20%	0	Retrospective, multicenter	Canada	Expert consensus	20.0	Exclusion of 19 undetermined cases, fever initially as dichotomous variable^c^

*Note*: All values shown after exclusion of undetermined/unclear and pHLH cases.

Abbreviations: HLH, hemophagocytic lymphohistiocytosis; ICU, intensive care unit; MAS, Macrophage activation syndrome; pHLH, primary hemophagocytic lymphohistiocytosis; sHLH, secondary hemophagocytic lymphohistiocytosis; sIL‐2R, soluble interleukin‐2 receptor.

^a^ Combined, other, or unknown trigger diseases are not considered.

^b^ Among the 15 requested variables.

^c^ For our analyses recoded as 38.3°C (fever) and 37.3°C (non‐fever) based on [18]. For calculation of HScore, febrile patients were grouped into 38.4–39.4°C category [9].

^d^ Converted into U/mL approximately using the factor 0.113 as suggested for the invitrogen ELISA (http://tools.thermofisher.com/content/sfs/manuals/MAN0014095_EH2IL2Rx_HusIL2R_ELISA_UG_PI.pdf).

^e^ Leukocytes extrapolated using the factor 1/ 1.67 [15].

### Validation of original, modified, and our newly developed HLH diagnostic criteria sets and biomarkers

Table 3 shows Youden's indices between original, modified, and our newly developed HLH diagnostic criteria sets as well as biomarkers for all validation cohorts. Corresponding quality criteria are shown in Table S10. Depending on Youden's index mean, the best performing HLH diagnostic criteria sets over all validation cohorts were the original HLH‐2004 criteria with a decreased cut‐off of 4 compared to the original cut‐off of 5 fulfilled HLH‐2004 criteria (mean sensitivity 86.5%, mean specificity 86.1%), followed by the revHLH‐2004 (4 fulfilled criteria as cut‐off, mean sensitivity 83.8%, mean specificity 87.8%) and the HScore (169 as cut‐off, mean sensitivity 82.4%, mean specificity 87.6%). Except for aHLH‐2004, revHLH‐2004 (4 fulfilled criteria as cut‐off), and the HScore (168 and 169 as cut‐offs), all diagnostic criteria sets and biomarkers performed inferior compared to the HLH‐2004 criteria (4 fulfilled criteria as cut‐off) in sign test, that is also HLH‐2004 criteria and revHLH‐2004 with the original cut‐offs of 5 fulfilled criteria. When only ICU cohorts were considered, revHLH‐2004 (3 fulfilled criteria as cut‐off, mean sensitivity 96.5%, mean specificity 86.4%), HScore (168 or 169 as cut‐off, mean sensitivity 86.5%, mean specificity 94.7%), original HLH‐2004 criteria with a cut‐off of 4 instead of the original 5 fulfilled criteria and revHLH‐2004 (both: 4 fulfilled criteria as cut‐off, mean sensitivity 77.2%, mean specificity 95.1%) showed best performance. Iteration 1 (5 fulfilled criteria as cut‐off, mean sensitivity 60.8%, mean specificity 95.6%) was the best of our newly developed HLH diagnostic criteria sets over all validation cohorts but showed inferior performance compared to the original and modified HLH‐2004 criteria as well as the HScore. Optimal ferritin cut‐offs ranged between 202 and 6843 µg/L, whereas 9083 µg/L as cut‐off showed moderate performance only in ICU cohorts (mean sensitivity 60.7%, mean specificity 97.6%). sIL‐2R and OHI index both had only low Youden's index mean. Our results were confirmed by sensitivity analyses for patients of at least 5 or 6 obtained HLH‐2004 criteria, respectively, and also for cohorts with HLH diagnosis based on expert review (Tables S5–S7).

**Table 3:** Validation of original, modified and our newly developed hemophagocytic lymphohistiocytosis (HLH) diagnostic criteria sets and biomarkers (only Youden's indices).

	Fulfilled criteria or cut‐off	Fardet et al. 2016 [9]	Debaugnies et al. 2016 [10]	Horrillo et al. 2019 [20]	Meena et al. 2020 [14]	Debaugnies et al. 2021 [19]	Lopez Marcos et al. 2021 [21]	Smits et al. 2021 [22]	Oh et al. 2021 [23]	Yao H et al. 2021 [24]	Yao S et al. 2021 [25]	Ammouri et al. 2022 [26]	He et al. 2022 [27]	Bilston et al. 2022 [12]	Youden's index mean	Youden's index mean (ICU cohorts)^a^	Validation cohorts performing worse than respective HLH‐2004 (cut‐off 4) [*n*, *p*‐value]^b^
HLH‐2004 [4]	4^c^	0.685	0.598	0.773	0.840	0.605	0.450	0.789	0.437	0.920	0.948	1.000	0.629	0.755	0.725	0.723	−
	5	0.462	0.514	0.542	0.588	0.491	0.442	0.732	0.588	0.768	0.817	0.850	0.370	0.577	0.595	0.540	12, 0.003
aHLH‐2004	4	0.682	0.542	0.593	0.768	0.615	0.480	0.742	0.566	0.929	0.885	0.950	0.472	0.760	0.691	0.692	8, 0.581
oHLH‐2004	4	0.607	0.551	0.389	0.684	0.543	0.391	0.699	0.655	0.875	0.846	0.700	0.417	0.702	0.620	0.614	12, 0.003
revHLH‐2004	3	0.511	0.558	0.188	0.832	0.825	0.284	0.568	0.299	0.500	0.947	0.979	0.628	0.699	0.601	0.829	12, 0.003
	4	0.685	0.598	0.773	0.840	0.605	0.450	0.773	0.541	0.830	0.919	1.000	0.534	0.755	0.716	0.723	4, 0.375
	5	0.462	0.514	0.542	0.589	0.491	0.442	0.689	0.478	0.607	0.721	0.850	0.274	0.577	0.557	0.540	12, 0.003
shHLH‐2004	3	0.545	0.550	0.295	0.748	0.539	0.257	0.552	0.240	0.920	0.945	0.979	0.690	0.656	0.609	0.644	11, 0.022
	4	0.544	0.571	0.383	0.663	0.605	0.396	0.716	0.357	0.705	0.804	1.000	0.417	0.572	0.595	0.634	11, 0.022
	5	0.247	0.390	0.114	0.198	0.500	0.345	0.503	0.168	0.393	0.490	0.450	0.205	0.203	0.324	0.349	13, <0.001
HScore [9]	168^c^	0.819	0.732	0.461	0.740	0.881	0.468	0.532	0.441	0.839	0.780	0.945	0.609	0.826	0.698	0.811	7, 1.000
	169^d^	0.819	0.703	0.475	0.740	0.881	0.541	0.532	0.441	0.848	0.751	0.950	0.595	0.829	0.700	0.811	7, 1.000
	optimal^e^	177; 0.824	168; 0.732	177; 0.538	167; 0.838	120; 0.896	171; 0.541	123; 0.644	164; 0.466	173; 0.911	106; 0.891	135; 0.963	151; 0.711	157; 0.869	–	–	–
Iteration 1	5	0.490	0.618	0.147	0.686	0.534	0.375	0.697	0.596	0.741	0.673	0.550	0.315	0.557	0.537	0.610	11, 0.022
	6	0.540	0.571	0.223	0.589	0.472	0.418	0.651	0.638	0.696	0.635	0.550	0.370	0.592	0.534	0.437	12, 0.003
Iteration 2	4	0.265	0.552	0.041	0.489	0.562	0.445	0.480	0.527	0.607	0.525	0.400	0.267	0.497	0.435	0.526	12, 0.003
Iteration 5	6	0.435	0.504	0.179	0.289	0.543	0.320	0.481	0.222	0.464	0.396	0.700	0.192	0.470	0.400	0.416	13, <0.001
Iteration 6	6	0.451	0.485	0.112	0.384	0.553	0.420	0.407	0.187	0.393	0.356	0.600	0.151	0.427	0.379	0.469	13, <0.001
Iteration 8	6	0.540	0.590	0.167	0.584	0.543	0.420	0.494	0.282	0.554	0.423	0.645	0.458	0.533	0.479	0.564	13, <0.001
Iteration 11	5	0.314	0.571	0.136	0.393	0.605	0.213	0.405	0.428	0.571	0.588	0.350	0.445	0.183	0.400	0.499	12, <0.001
Iteration 12	5	0.191	0.524	0.037	0.291	0.562	0.364	0.467	0.243	0.411	0.467	0.350	0.164	0.312	0.337	0.427	13, <0.001
	6	0.315	0.457	0.199	0.295	0.553	0.376	0.627	0.304	0.571	0.496	0.650	0.301	0.498	0.434	0.424	13, <0.001
Iteration 13	5	0.237	0.590	0.231	0.486	0.472	0.231	0.492	0.301	0.304	0.433	0.250	0.164	0.278	0.344	0.479	13, <0.001
	6	0.327	0.418	0.155	0.289	0.553	0.411	0.567	0.269	0.518	0.462	0.550	0.274	0.489	0.406	0.421	13, <0.001
	7	0.198	0.485	0.052	0.195	0.491	0.414	0.408	0.268	0.321	0.365	0.450	0.151	0.294	0.315	0.343	13, <0.001
Ferritin [µg/L]	9083^c^	0.306	0.335	0.143	0.461	0.705	0.073	0.152	0.313	0.438	0.214	0.300	0.043	0.545	0.310	0.583	12, 0.003
	optimal^e^	1985; 0.642	3353; 0.429	1351; 0.270	1197; 0.601	1799; 0.740	802; 0.482	1140; 0.383	5061; 0.430	9574; 0.464	481; 0.721	692; 1.000	202; 0.688	6843; 0.647	–	–	–
sIL‐2R [U/mL]	4621^c^	n.a.	0.050	n.a.	n.a.	0.286	0.333	0.312	0.284	0.042	0.327	n.a.	0.120	0.192	0.206	0.286	9, 0.004
	optimal^e^	n.a.	462; 0.350	n.a.	n.a.	576; 0.635	2849; 0.556	1171; 0.524	1829; 0.443	847; 0.813	753; 0.715	n.a.	740; 0.495	4285; 0.229	–	–	–
OHI index [11]	sIL‐2R > 3900 U/mL and ferritin > 1000 µg/L	n.a.	0.050	n.a.	n.a.	0.286	0.333	0.324	0.315	0.104	0.366	n.a.	0.162	0.175	0.235	0.286	9, 0.004

*Note*: Results shown as Youden's indices. Corresponding quality criteria (area under the curve [%], accuracy [%], sensitivity [%], specificity [%], positive predictive value [%], and negative predictive value [%]) presented in Table S10.

Abbreviations: aHLH‐2004, adjusted HLH‐2004 criteria; HLH, Hemophagocytic Lymphohistiocytosis; n.a., not applicable; OHI, optimized HLH inflammatory; oHLH‐2004, optimized HLH‐2004 criteria; revHLH‐2004, revised HLH‐2004 criteria; shHLH‐2004, shortened HLH‐2004 criteria; sIL‐2R, soluble interleukin‐2 receptor.

^a^Only Meena et al. [14] and Debaugnies et al. [19] considered.

^b^Statistical analyses performed using sign test.

^c^ Optimal cut‐off in our previous study [15].

^d^ Optimal cut‐off within the developmental dataset [9].

^e^ Optimal cut‐off determined for each study, shown with corresponding Youden's indices.

### Evaluation of ferritin as HLH screening marker

Six out of 13 validation cohorts reported HLH patients with ferritin < 500 µg/L (“Ferritin‐negative HLH”), which showed rates between 1.3% and 34.8% (Table 2). None of the ICU cohorts comprised ferritin‐negative HLH patients. Sensitivities of different ferritin cut‐offs to diagnose HLH are shown in Table S11. Highest ferritin cut‐offs with sensitivity of 100% ranged between 15 and 1018 µg/L over all validation cohorts, as well as between 533 and 794 µg/L over ICU cohorts. A ferritin cut‐off of 500 µg/L showed high mean sensitivity over all (94.0%) and ICU (100%) cohorts. When ferritin cut‐off was decreased to 400 or 300 µg/L, mean sensitivity slightly increased over all cohorts (95.4% and 97.0%). Raising ferritin cut‐off to 1000 or 3000 µg/L markedly decreased mean sensitivity over all and ICU cohorts, respectively.

## Discussion

This is the first multicenter study that aimed to optimize and validate diagnostic criteria in heterogeneous cohorts of secondary HLH patients. We developed new sets of criteria, which showed excellent performances in our cohort of 2623 adult critically ill patients and validated these together with the original and modified HLH‐2004 criteria as well as the HScore in 13 different cohorts of secondary HLH patients, which were identified by a systematic literature search and which we received original data on. We found that the original HLH‐2004 criteria with a decreased cut‐off of four fulfilled criteria was the best performing HLH diagnostic criteria set over all validation cohorts, followed by revHLH‐2004 and an HScore of 169 as a cut‐off. In the two ICU cohorts, revHLH‐2004, HScore, and original HLH‐2004 criteria showed the best performance. Our newly developed HLH diagnostic criteria, as well as sIL‐2R and OHI index, performed inferior over all validation cohorts. Optimal ferritin to diagnose HLH showed a wide range between the cohorts. A ferritin cut‐off of 500 µg/L had 94.0% mean sensitivity over all cohorts.

In previous analyses, we could demonstrate that fulfilling four HLH‐2004 criteria is optimal for HLH diagnosis in critically ill patients [15]. HLH‐2004 criteria have originally been adopted from pediatric populations without having been validated in an adult cohort. In the original HLH‐2004 criteria, HLH is formally diagnosed with five positive out of eight criteria. In the present study, we performed a multicenter validation of the HLH‐2004 criteria with satisfying mean sensitivity (86.5%) and mean specificity (86.1%) over both ICU and non‐ICU cohorts for a decreased cut‐off of 4 compared to the original cut‐off of 5 fulfilled HLH‐2004 criteria, as currently recommended [3]. The better overall performance, according to Youden's index, suggests that the considerable increase in sensitivity is not outweighed by the decrease in specificity, compared to a cut‐off of 5. The importance of early detection and the consequences of intervening, if not necessary, may, of course, be very dependent on the clinical situation and the condition of interest.

Omission of NK cell activity resulted in only minor worse results compared to the original HLH‐2004 criteria (4 fulfilled criteria as cut‐off, mean sensitivity 83.8%, mean specificity 87.8%). This is in accordance with the recently revHLH‐2004 for the diagnosis of pHLH, where removing NK cell activity led to a slight decrease in sensitivity and a slight increase in specificity, at least for a cut‐off of 5 fulfilled criteria [5]. The revHLH‐2004 requires 5 fulfilled criteria to diagnose pHLH, whereas we found a cut‐off of 4 better performing for diagnosis of secondary HLH.

A total of 13 cohorts included in our analyses comprised both children and adult secondary HLH patients with hematologic malignancy (e.g., lymphoma), rheumatologic disease (e.g., adult‐onset Still's disease, systemic lupus erythematosus) and infections such as visceral leishmaniasis and Epstein–Barr virus infection. Until now, five fulfilled criteria were always recommended, but our current systematic validation provides support for a secondary HLH diagnosis as soon as the cut‐off of 4 is reached. This will lead to a more rapid diagnosis by easily available criteria with consecutive improved outcomes. Timely diagnosis is crucial for survival [28]; thus, mortality may be further reduced.

Considering only ICU cohorts, the HLH‐2004 criteria with a cut‐off of 4 performed slightly worse (mean sensitivity 77.2%, mean specificity 95.1%) than in the entire cohort. Still, overall they were very good, which is why they can also be used safely in ICU patients. Remarkably, the revHLH‐2004 (without NK cell activity) with a cut‐off of 3 performed best over the ICU cohorts (mean sensitivity 96.5%, mean specificity 86.4%). However, we would interpret these findings cautiously as in our experience, three positive criteria can be reached rather quickly without HLH being present. Moreover, only two sole ICU cohorts were considered.

The results of the original HLH‐2004 criteria also performed better when compared to the modified HLH‐2004 criteria, albeit narrowly, as well as to our newly developed criteria. The inferior performance of our newly developed criteria may be due to being developed in our very specific, severely ill cohort at a tertiary care center, and also due to overfitting bias as we only have 40 HLH patients in our large cohort of 2623 patients. Whether they show good performance in other cohorts of severely ill patients needs further investigation. Additionally, further studies should investigate better performing diagnostic criteria by an improved statistical method, a larger dataset of several less heterogeneous cohorts, and an advanced machine learning approach.

In addition to the HLH‐2004 criteria, we were also able to validate the HScore, although the HScore was developed in a retrospective cohort with almost no patients having autoimmune/autoinflammatory triggered HLH [9]. The present 13 cohorts with a diverse patient population provided a good basis for validating the HScore and the HLH‐2004 criteria for a broad population of children and adult patients. Although HLH‐2004 criteria performed in some extent better than the HScore in the total cohort, the HScore cut‐off of 169 still showed a good mean sensitivity (82.4%) and mean specificity (87.6%). In the ICU cohorts, it was even slightly better (mean sensitivity 86.5%, mean specificity 94.7%) than the original HLH‐2004 criteria with a reduced cut‐off of 4. It can therefore also be used safely to diagnose secondary HLH.

In our previous study of critically ill patients [16], we had identified a ferritin cut‐off of 9083 µg/L with a sensitivity of 92.5% and a specificity of 91.9% as best predicting for secondary HLH diagnosis in critically ill patients. Within the validation cohorts, optimal ferritin cut‐offs varied widely between 202 and 6843 µg/L. The cut‐off of 9083 µg/L performed inferior in the overall cohort, however, slightly better in the ICU cohorts (mean sensitivity 60.7%, mean specificity 97.6%). It may be hypothesized that the more severely ill the patients are, the higher the respective ferritin cut‐off will be. Therefore, according to our results, ferritin alone may not be used for HLH diagnosis but proved as a reliable HLH screening marker using the cut‐off of 500 µg/L from the original HLH‐2004 criteria, of which we showed good mean sensitivity over all (94.0%) and ICU (100%) cohorts, respectively. Interestingly, ferritin‐negative HLH (ferritin < 500 µg/L) was seen in six cohorts with ratios between 1.3% and 34.8%. It has previously been reported that 10% of HLH patients present with ferritin < 500 µg/L [29]. Importantly, in daily practice, HLH in patients without elevated ferritin also needs consideration. Unfortunately, sIL‐2R was only determined in nine cohorts. Overall, it performed also inferior in our analysis, just like the recently published OHI index, which, however, showed good performance for the detection of hematologic malignancy‐associated HLH in a recent study [11]. Therefore, both sIL‐2R and OHI index should neither be used for the sole diagnosis of secondary HLH nor as an HLH screening parameter, at least in general patient populations. However, in more specific populations and based on unique triggers, MAS criteria [30], macrophage activation‐like syndrome criteria [31], immune‐effector‐cell‐associated HLH criteria [32], as well as the OHI index [11] may be valuable early indicators for the most specific diagnosis and therapy possible.

According to current recommendations, HLH should be diagnosed in conjunction with clinical judgment and patient history [3]. We therefore recommend that an assessment of the need for HLH‐specific therapy should be focused on the clinical course, that is, a patient who meets the criteria but does not improve despite adequate anti‐infective treatment is very likely to require HLH‐specific therapy. On the other hand, patients fulfilling HLH‐2004 criteria who improve clinically by adequate trigger treatment usually do not require HLH therapy. Therefore, the question should not be whether the patient has HLH, but whether the patient needs HLH‐specific therapy.

Several limitations of the present study deserve consideration. First of all, this is a retrospective analysis of existing data. Only 3 [10, 12, 14] out of the 13 validation cohorts studied the performance of the HLH‐2004 criteria. However, in each of these studies, a cut‐off of 4 fulfilled HLH‐2004 criteria also performed better compared to 5 fulfilled HLH‐2004 criteria. Second, as HLH is a rare disease, our dataset was highly unbalanced, with 40 HLH and 2583 non‐HLH patients, leading to a high risk of overfitting bias. Third, we depended on the authors to provide their data. Unfortunately, 151 did not reply or send any data; only 4 had incomplete datasets (more than 31% of missing values). Missing data may also have contributed to bias: the overall missing data rate varied from 1.6% to 30.0% between the 13 validation cohorts, with partially missing complete parameters. However, our results were supported by sensitivity analyses for patients of at least five or six obtained HLH‐2004 criteria, respectively. Fourth, of the 13 cohorts included, only 4 were prospective studies. Furthermore, the datasets provided were markedly heterogeneous in terms of ages, underlying diseases, patient populations, inclusion criteria, HLH prevalence, regions, and what “standard” is used to adjudicate HLH diagnosis (HLH‐2004 criteria versus expert consensus). Moreover, the distributions of variables were rather different. This fact might limit the applicability of our results. However, including heterogeneous patient cohorts for validation ensures that a broad spectrum of patients is represented. Finally, the diagnosis of HLH was based on the HLH‐2004 criteria in seven cohorts, which, of course, may have biased our results towards a favorable performance of the HLH‐2004 criteria. In addition, validation of HLH diagnostic criteria was done based on HLH patients, who were initially diagnosed by then unvalidated HLH‐2004 criteria, at least partially, when no HLH expert review was performed. However, our results were confirmed by a sensitivity analysis for cohorts with HLH diagnosis based on expert review, which should have reduced this bias.

## Conclusions

In this first multicenter validation study, we found that the original HLH‐2004 criteria with a reduced cut‐off of 4 compared to the original and recommended cut‐off of 5 fulfilled HLH‐2004 criteria were the best performing HLH diagnostic criteria set over all validation cohorts (mean sensitivity 86.5%, mean specificity 86.1%), followed by the revHLH‐2004 (4 fulfilled criteria as cut‐off, mean sensitivity 83.8%, mean specificity 87.8%) and the HScore (169 as cut‐off, mean sensitivity 82.4%, mean specificity 87.6%). Our newly developed HLH diagnostic criteria showed inferior performance over all validation cohorts, which might be due to the very specific developmental cohort of most severely ill adult patients. Overall, we found a new cut‐off of the HLH‐2004 criteria, which will lead to rapid diagnosis and improved patient outcomes. Four fulfilled HLH‐2004 criteria and an HScore of 169 were suitable to diagnose secondary HLH. A ferritin cut‐off of 500 µg/L proved as a reliable HLH screening marker. Given that the present study is the first formal validation of the HLH‐2004 criteria in secondary HLH, our results should be taken into account in clinical recommendations and in designing new studies.

## Author contributions

*Conceived and designed the study*: Gunnar Lachmann, Patrick Heeren, Cornelia Lachmann. *Obtained the data (own cohort)*: Gunnar Lachmann, Friederike S. Schuster, Peter Nyvlt, Thomas Schenk, Cornelia Lachmann. *Literature Search*: Gunnar Lachmann, Cornelia Lachmann. *Provided the data (validation cohorts)*: Wafa Ammouri, France Debaugnies, Lionel Galicier, Yuan Jia, Carole Nagant, Olaf Neth, Stefan Nierkens, Nikhil Meena, Juan San Martin, Hao Wei (Linda) Sun, Yini Wang, Zhao Wang, Jae‐Ho Yoon. *Data curation*: Gunnar Lachmann, Patrick Heeren, Cornelia Lachmann. *Software*: Patrick Heeren. *Analyzed the data*: Gunnar Lachmann, Patrick Heeren, Friederike S. Schuster, Cornelia Lachmann. *Supervision*: Gunnar Lachmann, Claudia Spies, Insa Feinkohl, Frank M. Brunkhorst, Paul La Rosée, Gritta Janka, Cornelia Lachmann. *Writing—original draft*: Gunnar Lachmann, Patrick Heeren, Cornelia Lachmann. *Writing—review and editing*: all authors.

## Conflict of interest statement

The authors declare no conflicts of interest.

## Funding information

Cornelia Lachmann is a participant in the Berlin Institute of Health (BIH) Charité Digital Clinician Scientist Program funded by the Charité—Universitätsmedizin Berlin and the BIH at Charité. Gunnar Lachmann was a participant in the BIH Charité Clinician Scientist Program funded by the Charité—Universitätsmedizin Berlin and the BIH at Charité.

## Data availability

The data that support the findings of this study are available on request from the corresponding author. The data are not publicly available due to privacy or ethical restrictions.

## Supporting information




**Table S1**. HLH‐2004 criteria to diagnose HLH based on Henter et al. [4].
**Table S2**. HScore based on Fardet et al. [9].
**Table S3**. Cut‐off ranges including 10 cut‐offs for each criterion, first step of exhaustive grid search method for cut‐off optimization.
**Table S4**. Narrowed down cut‐off ranges including 10 cut‐offs for each criterion, third step of exhaustive grid search method for cut‐off optimization.
**Table S5**. Validation of original, modified and our newly developed HLH diagnostic criteria sets and biomarkers (Sensitivity analyses for patients of at least 5 obtained HLH‐2004 criteria).
**Table S6**. Validation of original, modified and our newly developed HLH diagnostic criteria sets and biomarkers (Sensitivity analyses for patients of at least 6 obtained HLH‐2004 criteria).
**Table S7**. Validation of original, modified and our newly developed HLH diagnostic criteria sets and biomarkers (Sensitivity analyses for cohorts with HLH diagnosis based on expert review).
**Table S8**. Best cut‐offs and quality criteria of HLH diagnostic criteria sets within our dataset.
**Table S9**. Detailed description of requested data and missing data rate of the validation cohorts.
**Table S10**. Validation of original, modified, and our newly developed HLH diagnostic criteria sets and biomarkers (with quality criteria).
**Table S11**. Sensitivities of different ferritin cut‐offs to diagnose HLH.

**Fig. S1**. Optimizing strategy.
