# Supplementary material for: Multicenter validation of secondary hemophagocytic lymphohistiocytosis diagnostic criteria
Source: J Intern Med. 2025 Jan 27;297(3):312–27. doi: 10.1111/joim.20065 (PMC11846073; doi:10.1111/joim.20065)
Supplement: Supplementary file 1 — Table S1. HLH‐2004 criteria to diagnose HLH based on Henter et al. [4]. Table S2. HScore based on Fardet et al. [9]. Table S3. Cut‐off ranges including 10 cut‐offs for each criterion, first step of exhaustive grid search method for cut‐off optimization. Table S4. Narrowed down cut‐off ranges including 10 cut‐offs for each criterion, third step of exhaustive grid search method for cut‐off optimization. Table S5. Validation of original, modified and our newly developed HLH diagnostic criteria sets and biomarkers (Sensitivity analyses for patients of at least 5 obtained HLH‐2004 criteria). Table S6. Validation of original, modified and our newly developed HLH diagnostic criteria sets and biomarkers (Sensitivity analyses for patients of at least 6 obtained HLH‐2004 criteria). Table S7. Validation of original, modified and our newly developed HLH diagnostic criteria sets and biomarkers (Sensitivity analyses for cohorts with HLH diagnosis based on expert review). Table S8. Best cut‐offs and quality criteria of HLH diagnostic criteria sets within our dataset. Table S9. Detailed description of requested data and missing data rate of the validation cohorts. Table S10. Validation of original, modified, and our newly developed HLH diagnostic criteria sets and biomarkers (with quality criteria). Table S11. Sensitivities of different ferritin cut‐offs to diagnose HLH. [file JOIM-297-312-s001.docx]

**Supplement**

**Supplement Table 1. HLH-2004 criteria to diagnose HLH based on Henter et al. [4].**

| **HLH-2004 criteria: molecular diagnosis consistent with HLH or ≥ 5 criteria must be fulfilled** |
| --- |
| - Ferritin ≥ 500 µg/L - Fever - Splenomegaly - Cytopenias in ≥ 2 lines (hemoglobin < 9 g/dL, thrombocytes < 100 /nL, neutrophils < 1.0 /nL) - Hypertriglyceridemia and/or hypofibrinogenemia  (fasting triglycerides > 265 mg/dL, fibrinogen < 1.5 g/L) - Hemophagocytosis in bone marrow or spleen or lymph nodes - Low or absent natural killer cell activity - Soluble IL-2 receptor ≥ 2,400 U/mL |

**Supplement Table 2. HScore based on Fardet et al. [9].**

| **HScore: calculated by scoring criteria of each parameter (http://saintantoine.aphp.fr/score/)** |
| --- |
| Known underlying immunosuppression (no \| yes)  Temperature (< 38.4 °C \| 38.4 - 39.4 °C \| > 39.4 °C)  Organomegaly (no \| hepato- or splenomegaly \| hepatosplenomegaly)  Cytopenias (1 \| 2 \| 3, hemoglobin ≤ 9.2 g/dL, leukocytes ≤ 5,000 /µL, thrombocytes ≤ 110,000 /µL)  Ferritin (< 2,000 µg/L \| 2,000 - 6,000 µg/L \| > 6,000 µg/L)  Triglycerides (< 1.5 mM \| 1.5 - 4 mM \| > 4 mM)  Fibrinogen (> 2.5 g/L \| ≤ 2.5 g/L)  Aspartate aminotransferase (< 30 U/L \| ≥ 30 U/L)  Hemophagocytosis in bone marrow aspirate (no \| yes) |

**Supplement Table 3. Cut-off ranges including 10 cut-offs for each criterion, first step of exhaustive grid search method for cut-off optimization.**

|  | Cut-off 1 | Cut-off 2 | Cut-off 3 | Cut-off 4 | Cut-off 5 | Cut-off 6 | Cut-off 7 | Cut-off 8 | Cut-off 9 | Cut-off 10 |
| --- | --- | --- | --- | --- | --- | --- | --- | --- | --- | --- |
| Ferritin [µg/L] (≥) | 3102 | 39167 | 75232 | 111297 | 147362 | 183427 | 219492 | 255557 | 291622 | 327687 |
| Core body temperature [°C] (≥) | 36.6 | 37.1 | 37.6 | 38.2 | 38.7 | 39.2 | 39.7 | 40.3 | 40.8 | 41.3 |
| Hemoglobin [g/dL] (<) | 4.8 | 5.3 | 5.7 | 6.2 | 6.7 | 7.1 | 7.6 | 8.1 | 8.5 | 9 |
| Thrombocytes [/nL] (<) | 1 | 18 | 35 | 52 | 69 | 85 | 102 | 119 | 136 | 153 |
| Leukocytes [/nL] (<) | 0.08 | 3.7 | 7.4 | 11.0 | 14.7 | 18.3 | 22.0 | 25.6 | 29.3 | 32.9 |
| Triglycerides [mg/dL] (>) | 110 | 243 | 376 | 509 | 642 | 776 | 909 | 1042 | 1175 | 1308 |
| Fibrinogen [g/L] (<) | 0.3 | 1.0 | 1.6 | 2.3 | 3.0 | 3.7 | 4.4 | 5.0 | 5.7 | 6.4 |
| sIL-2R [U/mL] (≥) | 687 | 6193 | 11699 | 17205 | 22711 | 28216 | 33722 | 39228 | 44734 | 50240 |

sIL-2R, soluble interleukin-2 receptor.

**Supplement Table 4. Narrowed down cut-off ranges including 10 cut-offs for each criterion, third step of exhaustive grid search method for cut-off optimization.**

|  | Cut-off 1 | Cut-off 2 | Cut-off 3 | Cut-off 4 | Cut-off 5 | Cut-off 6 | Cut-off 7 | Cut-off 8 | Cut-off 9 | Cut-off 10 |
| --- | --- | --- | --- | --- | --- | --- | --- | --- | --- | --- |
| Ferritin [µg/L] (≥) | 3102 | 4147 | 5190 | 6235 | 7279 | 8323 | 9367 | 10412 | 11456 | 12500 |
| Core body temperature [°C] (≥) | 37.9 | 38.0 | 38.1 | 38.2 | 38.3 | 38.4 | 38.5 | 38.6 | 38.7 | 38.8 |
| Hemoglobin [g/dL] (<) | 7.0 | 7.2 | 7.3 | 7.5 | 7.7 | 7.8 | 8.0 | 8.2 | 8.3 | 8.5 |
| Thrombocytes [/nL] (<) | 50 | 58 | 66 | 73 | 81 | 89 | 97 | 104 | 112 | 120 |
| Leukocytes [/nL] (<) | 0.08 | 1.0 | 2.0 | 2.9 | 3.8 | 4.8 | 5.7 | 6.6 | 7.6 | 8.5 |
| Triglycerides [mg/dL] (>) | 110 | 221 | 332 | 443 | 554 | 666 | 777 | 888 | 999 | 1110 |
| Fibrinogen [g/L] (<) | 1.5 | 1.8 | 2.2 | 2.5 | 2.8 | 3.2 | 3.5 | 3.8 | 4.2 | 4.5 |
| sIL-2R [U/mL] (≥) | 687 | 1027 | 1367 | 1706 | 2046 | 2386 | 2726 | 3065 | 3405 | 3745 |
| ASAT [U/L]* (>) | 37 | 866 | 1695 | 2525 | 3354 | 4183 | 5012 | 5842 | 6671 | 7500 |

*Cut-off range created considering the distribution of the respective values of HLH and non-HLH patients, and the clinical meaningfulness. ASAT, aspartate aminotransferase. sIL-2R, soluble interleukin-2 receptor.

**Supplement Table 5. Validation of original, modified and our newly developed HLH diagnostic criteria sets and biomarkers (Sensitivity analyses for patients of at least 5 obtained HLH-2004 criteria).**

|  | Fulfilled criteria or cut-off | Fardet et al., 2016 [9] | Debaugnies et al., 2016 [10] | Horrillo et al., 2019 [20] | Meena et al., 2020 [14] | Debaugnies et al., 2021 [19] | Lopez Marcos et al., 2021 [21] | Smits et al., 2021 [22] | Oh et al., 2021 [23] | Yao H et al., 2021 [24] | Yao S et al., 2021 [25] | Ammouri et al., 2022 [26] | He et al., 2022 [27] | Bilston et al., 2022 [12] | Youden’s index mean | Youden’s index mean (ICU cohorts)**** | Validation cohorts performing worse than respective HLH-2004 (cut-off 4) [n, p-value]***** |
| --- | --- | --- | --- | --- | --- | --- | --- | --- | --- | --- | --- | --- | --- | --- | --- | --- | --- |
| HLH-2004 [4] | 4* | 90.4, 84.5, 85.8, 82.4, 88.5, 78.5, 0.682 | 85.8, 75.7, 77.1, 75.0, 61.4, 86.4, 0.521 | 93.2, 86.2, 97.6, 79.1, 74.5, 98.1, 0.767 | 96.6, 91.4, 90.0, 91.5, 25.7, 99.6, 0.815 | 97.2 92.8, 69.2, 95.9, 69.2, 95.9, 0.651 | 82.9, 66.7, 90.3, 51.1, 54.9, 88.9, 0.414 | 94.7, 89.0, 92.9, 85.5, 85.5, 92.9, 0.784 | 83.3, 75.2, 90.0, 53.7, 74.0, 78.6, 0.437 | 98.9, 95.2, 98.2, 93.8, 88.7, 99.1, 0.920 | 99.9, 98.3, 95.2, 99.6, 99.0, 98.0, 0.948 | 100,  100,  100, 100, 100, 100, 1.000 | 89.4, 79.2, 71.2, 91.5, 92.9, 67.2, 0.627 | 94.9, 91.1, 84.5, 91.9, 55.8, 98.0, 0.764 | 0.718 | 0.733 | - |
|  | 5 | 90.4, 67.4, 48.1, 98.0, 97.5, 54.3, 0.462 | 85.8, 81.6, 54.3, 95.6, 86.4, 80.2, 0.499 | 93.2, 81.7, 57.1, 97.0, 92.3, 78.3, 0.542 | 96.6, 97.1, 60.0, 98.4, 54.5, 98.7, 0.584 | 97.2, 93.7, 53.8, 99.0, 87.5, 94.2, 0.528 | 82.9, 78.2, 58.1, 91.5, 81.8, 76.8, 0.496 | 94.7, 87.0, 75.7, 97.4, 96.4, 81.3, 0.731 | 83.3, 79.2, 78.3, 80.5, 85.5, 71.7, 0.588 | 98.9, 92.3, 76.8, 100, 100, 89.6, 0.768 | 99.9, 94.5, 81.7, 100, 100, 92.8, 0.817 | 100, 98.6, 85.0, 100, 100, 98.4, 0.850 | 89.4, 61.7, 37.0, 100, 100, 50.5, 0.370 | 94.9, 94.3, 59.8, 98.5, 82.9, 95.3, 0.583 | 0.601 | 0.556 | 11, 0.022 |
| aHLH-2004 | 4 | 91.1, 82.6, 77.8, 90.2, 92.6, 71.9, 0.680 | 83.9, 77.7, 65.7, 83.8, 67.6, 82.6, 0.495 | 85.3, 80.7, 73.8, 85.1, 75.6, 83.3, 0.589 | 95.3, 94.9, 80.0, 95.4, 36.4, 99.3, 0.754 | 97.3, 93.7, 69.2, 96.9, 75.0, 96.0, 0.662 | 80.6, 73.1, 80.6, 68.1, 62.5, 84.2, 0.487 | 92.9, 87.0, 84.3, 89.5, 88.1, 86.1, 0.738 | 85.9, 80.2, 88.3, 68.3, 80.3, 80.0, 0.566 | 98.8, 95.8, 98.2, 94.6, 90.2, 99.1, 0.929 | 99.8, 96.6, 88.5, 100, 100, 95.3, 0.885 | 100, 99.5, 95.0, 100, 100, 99.5, 0.950 | 84.3, 69.2, 53.4, 93.6, 92.9, 56.4, 0.470 | 96.6, 93.9, 81.4, 95.4, 68.1, 97.7, 0.768 | 0.690 | 0.708 | 8, 0.581 |
| oHLH-2004 | 4 | 92.2, 76.5, 63.6, 97.1, 97.2, 62.7, 0.765 | 84.1, 78.6, 65.7, 85.3, 69.7, 82.9, 0.510 | 82.0, 74.3, 47.6, 91.0, 76.9, 73.5, 0.387 | 96.7, 96.8, 70.0, 97.7, 50.0, 99.0, 0.677 | 97.2, 92.8, 61.5, 96.9, 72.7, 95.0, 0.585 | 78.6, 74.4, 61.3, 83.0, 70.4, 76.5, 0.443 | 91.3, 84.9, 80.0, 89.5, 87.5, 82.9, 0.695 | 86.0, 83.2, 85.0, 80.5, 86.4, 78.6, 0.655 | 98.6, 95.2, 89.3, 98.2, 96.2, 94.8, 0.875 | 99.7, 95.4, 84.6, 100, 100, 93.8, 0.846 | 96.4, 97.1, 70.0, 100, 100, 96.9, 0.700 | 83.4, 65.8, 47.9, 93.6, 92.1, 53.7, 0.416 | 96.9, 94.3, 74.2, 96.7, 73.5, 96.9, 0.710 | 0.636 | 0.631 | 10, 0.092 |
| revHLH-2004 | 3 | 90.4, 79.9, 96.3, 53.9, 76.8, 90.2, 0.502 | 85.8, 63.1, 94.3, 47.1, 47.8, 94.1, 0.413 | 93.2, 48.6, 100, 16.4, 42.9, 100, 0.164 | 96.6, 77.5, 100, 76.7, 12.3, 100, 0.767 | 97.2, 90.1, 100, 88.8, 54.2, 100, 0.888 | 82.9, 48.7, 100, 14.9, 43.7, 100, 0.149 | 95.0, 77.4, 98.6, 57.9, 68.3, 97.8, 0.565 | 81.8, 70.3, 93.3, 36.6, 68.3, 78.9, 0.299 | 95.5, 69.0, 98.2, 54.5, 51.9, 98.4, 0.527 | 99.5, 97.4, 97.1, 97.5, 94.4, 98.8, 0.947 | 100, 98.1, 100, 97.9, 83.3, 100, 0.979 | 89.3, 80.8, 79.5, 83.0, 87.9, 72.2, 0.624 | 94.9, 77.2, 95.9, 75.0, 31.7, 99.3, 0.708 | 0.579 | 0.828 | 12, 0.003 |
|  | 4 | 90.4, 84.5, 85.8, 82.4, 88.5, 78.5, 0.682 | 85.8, 75.7, 77.1, 75.0, 61.4, 86.4, 0.521 | 93.2, 86.2, 97.6, 79.1, 74.5, 98.1, 0.767 | 96.6, 91.4, 90.0, 91.5, 25.7, 99.6, 0.815 | 97.2, 92.8, 69.2, 95.9, 69.2, 95.9, 0.651 | 82.9, 66.7, 90.3, 51.1, 54.9, 88.9, 0.414 | 95.0, 88.4, 90.0, 86.8, 86.3, 90.4, 0.768 | 81.8, 78.2, 83.3, 70.7, 80.6, 74.4, 0.541 | 95.5, 92.3, 89.3, 93.8, 87.7, 94.6, 0.830 | 99.5, 97.4, 92.3, 99.6, 99.0, 96.8, 0.919 | 100, 100, 100, 100, 100, 100, 1.000 | 89.3, 72.5, 57.5, 95.7, 95.5, 59.2, 0.533 | 94.9, 91.1, 84.5, 91.9, 55.8, 98.0, 0.764 | 0.708 | 0.733 | 4, 0.375 |
|  | 5 | 90.4, 67.4, 48.1, 98.0, 97.5, 54.3, 0.462 | 85.8, 81.6, 54.3, 95.6, 86.4, 80.2, 0.499 | 93.2, 81.7, 57.1, 97.0, 92.3, 78.3, 0.542 | 96.6, 97.1, 60.0, 98.4, 54.5, 98.7, 0.584 | 97.2, 93.7, 53.8, 99.0, 87.5, 94.2, 0.528 | 82.9, 78.2, 58.1, 91.5, 81.8, 76.8, 0.496 | 95.0, 84.9, 71.4, 97.4, 96.2, 78.7, 0.688 | 81.8, 71.3, 60.0, 87.8, 87.8, 60.0, 0.478 | 95.5, 86.9, 60.7, 100, 100, 83.6, 0.607 | 99.5, 91.7, 72.1, 100, 100, 89.4, 0.721 | 100, 98.6, 85.0, 100, 100, 98.4, 0.850 | 89.3, 55.8, 27.4, 100, 100, 47.0, 0.274 | 94.9, 94.3, 59.8, 98.5, 82.9, 95.3, 0.583 | 0.562 | 0.556 | 11, 0.022 |
| shHLH-2004 | 3 | 87.1, 79.9, 90.1, 63.7, 79.8, 80.2, 0.538 | 84.0, 68.9, 80.0, 63.2, 52.8, 86.0, 0.432 | 74.9, 56.0, 97.6, 29.9, 46.6, 95.2, 0.275 | 92.8, 79.4, 90.0, 79.0, 12.3, 99.6, 0.690 | 93.9, 86.5, 69.2, 88.8, 45.0, 95.6, 0.580 | 77.6, 48.7, 100, 14.9, 43.7, 100, 0.149 | 91.8, 76.7, 95.7, 59.2, 68.4, 93.8, 0.549 | 71.4, 66.3, 85.0, 39.0, 67.1, 64.0, 0.240 | 90.6, 68.5, 94.6, 55.4, 51.5, 95.4, 0.500 | 99.4, 97.7, 96.2, 98.4, 96.2, 98.4, 0.945 | 100, 98.1, 100, 97.9, 83.3, 100, 0.979 | 90.4, 83.3, 79.5, 89.4, 92.1, 73.7, 0.688 | 90.3, 77.5, 90.7, 75.8, 31.3, 98.5, 0.666 | 0.556 | 0.635 | 12, 0.003 |
|  | 4 | 87.1, 73.5, 61.1, 93.1, 93.4, 60.1, 0.542 | 84.0, 79.6, 65.7, 86.8, 71.9, 83.1, 0.525 | 74.9, 71.6, 57.1, 80.6, 64.9, 75.0, 0.377 | 92.8, 94.0, 70.0, 94.8, 30.4, 99.0, 0.648 | 93.9, 92.8, 69.2, 95.9, 69.2, 95.9, 0.651 | 77.6, 64.1, 83.9, 51.1, 53.1, 82.8, 0.349 | 91.8, 85.6, 84.3, 86.8, 85.5, 85.7, 0.711 | 71.4, 67.3, 65.0, 70.7, 76.5, 58.0, 0.357 | 90.6, 88.7, 75.0, 95.5, 89.4, 88.4, 0.705 | 99.4, 94.0, 80.8, 99.6, 98.8, 92.4, 0.804 | 100, 100, 100, 100, 100, 100, 1.000 | 90.4, 65.0, 43.8, 97.9, 97.0, 52.9, 0.417 | 90.3, 89.8, 64.9, 92.9, 52.5, 95.6, 0.578 | 0.590 | 0.650 | 10, 0.012 |
|  | 5 | 87.1, 53.8, 24.7, 100, 100, 45.5, 0.247 | 84.0, 78.6, 40.0, 98.5, 93.3, 76.1, 0.385 | 74.9, 65.1, 14.3, 97.0, 75.0, 64.4, 0.113 | 92.8, 97.1, 20.0, 99.7, 66.7, 97.4, 0.197 | 93.9, 94.6, 53.8, 100, 100, 94.2, 0.538 | 77.6, 74.4, 45.2, 93.6, 82.4, 72.1, 0.388 | 91.8, 76.0, 52.9, 97.4, 94.9, 69.2, 0.502 | 71.4, 51.5, 21.7, 95.1, 86.7, 45.3, 0.168 | 90.6, 79.8, 39.3, 100, 100, 76.7, 0.393 | 99.4, 84.8, 49.0, 100, 100, 82.2, 0.490 | 100, 94.7, 45.0, 100, 100, 94.5, 0.450 | 90.4, 51.7, 20.5, 100, 100, 44.8, 0.205 | 90.3, 90.5, 21.6, 98.9, 70.0, 91.2, 0.205 | 0.329 | 0.368 | 13, < 0.001 |
| HScore [9] | 168* | 96.8, 92.0, 96.3, 85.3, 91.2, 93.5, 0.816 | 89.0, 78.6, 91.4, 72.1, 62.7, 94.2, 0.635 | 83.1, 68.8, 88.1, 56.7, 56.1, 88.4, 0.448 | 93.8, 91.1, 80.0, 91.5, 23.5, 99.3, 0.715 | 97.7, 95.5, 100, 94.9, 72.2, 100, 0.949 | 80.7, 65.4, 83.9, 53.2, 54.2, 83.3, 0.371 | 91.0, 77.4, 55.7, 97.4, 95.1, 70.5, 0.531 | 78.5, 72.3, 73.3, 70.7, 78.6, 64.4, 0.441 | 99.0, 89.9, 98.2, 85.7, 77.5, 99.0, 0.839 | 98.8, 93.1, 78.8, 99.2, 97.6, 91.7, 0.780 | 99.8, 99.0, 95.0, 99.5, 95.0, 99.5, 0.945 | 90.8, 77.5, 67.1, 93.6, 94.2, 64.7, 0.607 | 97.5, 93.4, 89.7, 93.9, 64.0, 98.7, 0.836 | 0.686 | 0.832 | 8, 0.581 |
|  | 169** | 96.8, 92.0, 96.3, 85.3, 91.2, 93.5, 0.816 | 89.0, 77.7, 88.6, 72.1, 62.0, 92.5, 0.606 | 83.1, 69.7, 88.1, 58.2, 56.9, 88.6, 0.463 | 93.8, 91.1, 80.0, 91.5, 23.5, 99.3, 0.715 | 97.7, 95.5, 100, 94.9, 72.2, 100, 0.949 | 80.7, 73.1, 80.6, 68.1, 62.5, 84.2, 0.487 | 91.0, 77.4, 55.7, 97.4, 95.1, 70.5, 0.531 | 78.5, 72.3, 73.3, 70.7, 78.6, 64.4, 0.441 | 99.0, 90.5, 98.2, 86.6, 78.6, 99.0, 0.848 | 98.8, 92.2, 76.0, 99.2, 97.5, 90.6, 0.751 | 99.8, 99.5, 95.0, 100, 100, 99.5, 0.950 | 90.8, 76.7 65.8, 93.6, 94.1, 64.7, 0.594 | 97.5, 93.6, 89.7, 94.1, 64.9, 98.7, 0.838 | 0.691 | 0.832 | 6, 1.000 |
| Iteration 1 | 5 | 92.2, 68.9, 50.0, 99.0, 98.8, 55.5, 0.490 | 86.8, 82.5, 68.6, 89.7, 77.4, 84.7, 0.583 | 78.1, 66.1, 19.0, 95.5, 72.7, 65.3, 0.146 | 96.4, 97.1, 70.0, 98.0, 53.8, 99.0, 0.680 | 97.1, 91.9, 61.5, 95.9, 66.7, 94.9, 0.575 | 78.0, 73.1, 58.1, 83.0, 69.2, 75.0, 0.410 | 90.6, 84.9, 78.6, 90.8, 88.7, 82.1, 0.694 | 84.0, 79.2, 76.7, 82.9, 86.8, 70.8, 0.596 | 98.8, 91.1, 75.0, 99.1, 97.7, 88.8, 0.741 | 99.5, 90.2, 67.3, 100, 100, 87.8, 0.673 | 98.2, 95.7, 55.0, 100, 100, 95.4, 0.550 | 84.0, 58.3, 31.5, 100, 100, 48.5, 0.315 | 94.9, 93.3, 58.8, 97.5, 74.0, 95.1, 0.563 | 0.540 | 0.628 | 11, 0.022 |
|  | 6 | 92.4, 72.0, 54.9, 99.0, 98.9, 58.0, 0.540 | 85.6, 79.6, 65.7, 86.8, 71.9, 83.1, 0.525 | 75.1, 67.9, 31.0, 91.0, 68.4, 67.8, 0.220 | 95.4, 97.1, 60.0, 98.4, 54.5, 98.7, 0.584 | 97.0, 91.9, 53.8, 96.9, 70.0, 94.1, 0.508 | 78.0, 75.6, 64.5, 83.0, 71.4, 78.0, 0.475 | 90.9, 82.9, 71.4, 93.4, 90.9, 78.0, 0.648 | 83.9, 63.8, 83.3, 80.5, 86.2, 76.7, 0.638 | 99.5, 89.9, 69.6, 100, 100, 86.8, 0.696 | 99.6, 89.1, 63.5, 100, 100, 86.5, 0.635 | 98.3, 95.7, 55.0, 100, 100, 95.4, 0.550 | 85.7, 61.7, 37.0, 100, 100, 50.5, 0.370 | 95.1, 94.1, 61.9, 98.0, 78.9, 95.5, 0.599 | 0.538 | 0.546 | 10, 0.092 |
| Iteration 2 | 4 | 87.7, 54.9, 26.5, 100, 100, 46.2, 0.265 | 83.6, 81.6, 60.0, 92.6, 80.8, 81.8, 0.526 | 60.6, 60.6, 14.3, 89.6, 46.2, 62.5, 0.038 | 93.2, 96.8, 50.0, 98.4, 50.0 98.4, 0.484 | 94.9, 94.6, 61.5, 99.0, 88.9, 95.1, 0.605 | 77.2, 75.6, 67.7, 80.9, 70.0, 79.2, 0.486 | 86.2, 74.7, 54.3, 93.4, 88.4, 68.9, 0.477 | 82.2, 52.7, 60.0, 92.7, 92.3, 61.3, 0.527 | 91.3, 85.7, 64.3, 96.4, 90.0, 84.4, 0.607 | 97.8, 85.6, 52.9, 99.6, 98.2, 83.2, 0.525 | 92.8, 94.2, 40.0, 100, 100, 94.0, 0.400 | 82.4, 55.8, 28.8, 97.9, 95.5, 46.9, 0.266 | 94.8, 92.7, 52.6, 97.6, 72.9, 94.4, 0.502 | 0.439 | 0.545 | 10, 0.092 |
| Iteration 5 | 6 | 90.2, 65.5, 44.4, 99.0, 98.6, 52.9, 0.435 | 85.0, 76.7, 60.0, 85.3, 67.7, 80.6, 0.453 | 66.8, 65.1, 31.1, 86.6, 59.1, 66.7, 0.175 | 92.0, 96.2, 30.0, 98.4, 37.5, 97.7, 0.284 | 96.7, 92.8, 61.5, 96.9, 72.7, 95.0, 0.585 | 75.7, 57.7, 74.2, 46.8, 47.9, 73.3, 0.210 | 89.0, 74.7, 55.7, 92.1, 86.7, 69.3, 0.478 | 72.8, 57.4, 41.7, 80.5, 75.8, 48.5, 0.222 | 97.7, 82.1, 46.4, 100, 100, 78.9, 0.464 | 96.4, 81.6, 40.4, 99.2, 95.5, 79.6, 0.396 | 99.2, 97.1, 70.0, 100, 100, 96.9, 0.700 | 85.1, 50.8, 19.2, 100, 100, 44.3, 0.192 | 93.2, 92.0, 50.5, 97.0, 67.1, 94.2, 0.475 | 0.390 | 0.435 | 13, < 0.001 |
| Iteration 6 | 6 | 91.5, 66.3, 45.1, 100, 100, 53.4, 0.451 | 84.4, 76.7, 57.1, 86.8, 69.0, 79.7, 0.439 | 63.1, 61.5, 28.6, 82.1, 50.0, 64.7, 0.107 | 91.8, 95.9, 40.0, 97.7, 36.4, 98.0, 0.377 | 95.5, 93.7, 61.5, 98.0, 80.0, 95.0, 0.595 | 75.7, 69.2, 74.2, 66.0, 59.0, 79.5, 0.402 | 89.4, 71.2, 45.7, 94.7, 88.9, 65.5, 0.405 | 71.0, 54.5, 33.3, 85.4, 76.9, 46.7, 0.187 | 96.6, 79.8, 39.3, 100, 100, 76.6, 0.393 | 97.8, 80.7, 35.6, 100, 100, 78.5, 0.356 | 99.8, 96.2, 60.0, 100, 100, 95.9, 0.962 | 85.5, 48.3, 15.1, 100, 100, 43.1, 0.151 | 92.4, 91.3, 46.4, 96.7, 63.4, 93.7, 0.431 | 0.404 | 0.486 | 13, < 0.001 |
| Iteration 8 | 6 | 89.0, 73.9, 63.6, 90.2, 91.2, 60.9, 0.538 | 85.0, 79.6, 68.6, 85.3, 70.6, 84.1, 0.539 | 62.1, 59.6, 50.0, 65.7, 47.7, 67.7, 0.157 | 93.6, 96.5, 60.0, 97.7, 46.2, 98.7, 0.577 | 96.3, 92.8, 61.5, 96.9, 72.7, 95.0, 0.585 | 74.0, 57.7, 93.5, 34.0, 48.3, 88.9, 0.276 | 87.2, 75.3, 55.7, 93.4, 88.6, 69.6, 0.491 | 72.7, 62.4, 55.0, 73.2, 75.0, 52.6, 0.282 | 98.2, 85.1, 55.4, 100, 100, 81.8, 0.554 | 96.5, 82.8, 42.3, 100, 100, 80.3, 0.423 | 97.0, 96.2, 65.0, 99.5, 92.9, 96.4, 0.645 | 85.3, 68.3, 52.1, 93.6, 92.7, 55.7, 0.457 | 93.4, 92.0, 57.7, 96.1, 64.4, 94.9, 0.539 | 0.466 | 0.581 | 12, 0.003 |
| Iteration 11 | 5 | 83.4, 58.3, 33.3, 98.0, 96.4, 48.1, 0.314 | 81.5, 79.6, 65.7, 86.8, 71.9, 83.1, 0.525 | 60.3, 62.4, 31.0, 82.1, 52.0, 65.5, 0.130 | 93.7, 97.1, 40.0, 99.0, 57.1, 98.1, 0.390 | 95.6, 92.8, 69.2, 95.9, 69.2, 95.9, 0.651 | 73.3, 65.4, 38.7, 83.0, 60.0, 67.2, 0.217 | 87.9, 71.2, 44.3, 96.1, 91.2, 65.2, 0.403 | 77.7, 68.3, 55.0, 87.8, 86.8, 57.1, 0.428 | 97.3, 85.7, 57.1, 100, 100, 82.4, 0.571 | 97.6, 87.4, 59.6, 99.2, 96.9, 85.2, 0.588 | 98.4, 93.8, 35.0, 100, 100, 93.5, 0.350 | 86.5, 66.7, 46.6, 97.9, 97.1, 54.1, 0.444 | 86.9, 90.3, 19.6, 98.9, 67.9, 91.0, 0.185 | 0.400 | 0.521 | 11, 0.006 |
| Iteration 12 | 5 | 85.4, 50.4, 19.1, 100, 100, 43.8, 0.191 | 85.8, 82.5, 54.3, 97.1, 90.5, 80.5, 0.513 | 69.5, 67.0, 28.6, 91.0, 66.7, 67.0, 0.196 | 95.2, 96.5, 30.0, 98.7, 42.9, 97.7, 0.287 | 97.3, 94.6, 61.5, 99.0, 88.9, 95.1, 0.605 | 74.4, 70.5, 54.8, 80.9, 65.4, 73.1, 0.357 | 86.1, 74.0, 54.3, 92.1, 86.4, 68.6, 0.464 | 77.5, 56.4, 31.7, 92.7, 86.4, 48.1, 0.243 | 94.2, 80.4, 41.1, 100, 100, 77.2, 0.411 | 97.4, 83.9, 47.1, 99.6, 98.0, 81.5, 0.467 | 92.6, 93.8, 35.0, 100, 100, 93.5, 0.350 | 81.6, 49.2, 16.4, 100, 100, 43.5, 0.164 | 92.3, 91.4, 33.0, 98.5, 72.7, 92.4, 0.315 | 0.351 | 0.446 | 13, < 0.001 |
|  | 6 | 90.3, 58.0, 31.5, 100, 100, 47.9, 0.315 | 84.0, 77.7, 51.4, 91.2, 75.0, 78.5, 0.426 | 69.5, 67.0 28.6, 91.0, 66.7, 67.0, 0.196 | 92.2, 97.1, 30.0, 99.3, 60.0, 97.7, 0.293 | 97.1, 93.7, 61.5, 98.0, 80.0, 95.1, 0.595 | 75.8, 61.5, 74.2, 53.2, 51.1, 75.8, 0.274 | 89.7, 81.5, 72.9, 89.5, 86.4, 78.2, 0.623 | 75.3, 61.4, 45.0, 85.4, 81.8, 51.5, 0.614 | 98.2, 85.7, 57.1, 100, 100, 82.4, 0.571 | 97.9, 84.8, 50.0, 99.6, 98.1, 82.4, 0.496 | 98.9, 96.6, 65.0, 100, 100, 96.4, 0.650 | 87.4, 57.5, 30.1, 100, 100, 48.0, 0.301 | 94.8, 92.9, 52.6, 97.7, 73.9, 94.4, 0.503 | 0.451 | 0.444 | 12, 0.003 |
| Iteration 13 | 5 | 86.5, 53.4, 24.7, 99.0, 97.6, 45.3, 0.237 | 82.7, 83.5, 62.9, 94.1, 84.6, 83.1, 0.570 | 65.6, 66.1, 40.5, 82.1, 58.6, 68.8, 0.226 | 94.8, 96.5, 50.0, 98.0, 45.5, 98.4, 0.965 | 95.6, 91.9, 53.8, 96.9, 70.0, 94.1, 0.508 | 73.2, 69.2, 35.5, 91.5, 73.3, 68.3, 0.270 | 86.5, 75.3, 54.3, 94.7, 90.5, 69.2, 0.490 | 76.7, 59.4, 35.0, 95.1, 91.3, 50.0, 0.301 | 93.0, 76.8, 30.4, 100, 100, 74.2, 0.304 | 98.6, 83.0, 43.3, 100, 100, 80.5, 0.433 | 97.8, 92.8, 25.0, 100, 100, 92.6, 0.250 | 87.1, 49.2, 16.4, 100, 100, 43.5, 0.164 | 91.8, 90.8, 29.9, 98.2, 67.4, 92.0, 0.281 | 0.385 | 0.737 | 11, 0.022 |
|  | 6 | 91.8, 58.7, 32.7, 100, 100, 48.3, 0.327 | 83.2, 75.7, 48.6, 89.7, 70.8, 77.2, 0.383 | 65.0, 64.2, 28.6, 86.6, 57.1, 65.9, 0.151 | 92.1, 96.2, 30.0, 98.4, 37.5, 97.7, 0.284 | 96.0, 93.7, 61.5, 98.0, 80.0, 95.0, 0.595 | 77.0, 70.5, 71.0, 70.2, 61.1, 78.6, 0.412 | 89.9, 78.8, 64.3, 92.1, 88.2, 73.7, 0.564 | 73.9, 58.4, 36.7, 90.2, 84.6, 49.3, 0.269 | 97.2, 83.9, 51.8, 100, 100, 80.6, 0.518 | 98.7, 83.9, 46.2, 100, 100, 81.3, 0.462 | 99.6, 95.7, 55.0, 100, 100, 95.4, 0.550 | 88.6, 55.8, 27.4, 100, 100, 47.0, 0.274 | 93.9, 92.9, 51.5, 97.9, 74.6, 94.3, 0.494 | 0.406 | 0.440 | 13, < 0.001 |
|  | 7 | 91.4, 50.8, 19.8, 100, 100, 44.0, 0.198 | 84.5, 78.6, 54.3, 91.2, 76.0, 79.5, 0.455 | 65.9, 62.4, 9.5, 95.5, 57.1, 62.7, 0.050 | 90.4, 96.8, 20.0, 99.3, 50.0, 97.4, 0.193 | 96.3, 93.7, 53.8, 99.0, 87.5, 94.2, 0.528 | 78.7, 73.1, 58.1, 83.0, 69.2, 75.0, 0.410 | 89.1, 71.2, 47.1, 93.4, 86.8, 65.7, 0.406 | 75.5, 57.4, 31.7, 95.1, 90.5, 48.8, 0.268 | 97.1, 77.4, 32.1, 100, 100, 74.7, 0.321 | 98.2, 81.0, 36.5, 100, 100, 78.7, 0.365 | 99.8, 94.7, 45.0, 100, 100, 94.5, 0.450 | 87.1, 48.3, 15.1, 100, 100, 43.1, 0.151 | 94.2, 91.4, 30.9, 98.7, 75.0, 92.2, 0.297 | 0.315 | 0.361 | 13, < 0.001 |
| Ferritin [µg/L] | 9083* | 86.5, 56.8, 34.0, 96.6, 94.5, 45.7, 0.306 | 75.7, 72.3, 42.9, 89.8, 71.4, 72.6, 0.327 | 65.0, 63.8, 23.8, 90.5, 62.5, 64.0, 0.143 | 82.9, 93.7, 50.0, 95.1, 25.0, 98.3, 0.451 | 93.3, 95.5, 69.2, 99.0, 90.0, 96.0, 0.682 | 75.5, 62.9, 11.1, 95.3, 60.0, 63.1, 0.065 | 69.8, 55.7, 27.5, 87.1, 70.4, 51.9, 0.146 | 73.7, 62.4, 48.3, 82.9, 80.6, 52.3, 0.313 | 80.0, 74.4, 64.3, 79.5, 61.0, 81.7, 0.438 | 90.0, 28.9, 21.4, 100, 100, 12.0, 0.214 | 100, 93.3, 30.0, 100, 100, 93.1, 0.300 | 88.4, 42.6, 4.3, 100, 100, 41.1, 0.043 | 90.3, 91.0, 59.8, 94.7, 58.0, 95.1, 0.545 | 0.306 | 0.567 | 12, 0.003 |
| sIL-2R [U/mL] | 4621* | n.a. | 71.0, 36.7, 5.0, 100, 100, 34.5, 0.050 | n.a. | n.a. | 87.6, 91.9, 30.8, 100, 100, 91.6, 0.308 | 61.1, 45.5, 33.3, 100, 100, 25.0, 0.333 | 81.3, 65.8, 37.9, 93.2, 84.6, 60.4, 0.312 | 72.0, 51.2, 37.5, 90.9, 92.3, 33.3, 0.284 | 89.6, 9.8, 4.2, 100, 100, 6.1, 0.042 | 90.4, 36.4, 32.7, 100, 100, 8.1, 0.327 | n.a. | 75.0, 46.5, 14.3, 97.7, 90.9, 41.7, 0.120 | 58.2, 57.9, 55.6, 63.6, 78.9, 36.8, 0.192 | 0.219 | 0.308 | 9, 0.004 |
| OHI index [11] | sIL-2R > 3900 U/mL and ferritin > 1000 µg/L | n.a. | 52.5, 32.1, 5.0, 100, 100, 29.6, 0.050 | n.a. | n.a. | 65.4, 91.9, 30.8, 100, 100, 91.6, 0.308 | 66.7, 45.5, 33.3, 100, 100, 25.0, 0.333 | 66.2, 64.9, 36.2, 96.2, 91.3, 58.0, 0.324 | 65.8, 53.5, 40.6, 90.9, 92.9, 34.5, 0.316 | 55.2, 15.7, 10.4, 100, 100, 6.5, 0.104 | 68.3, 40.2, 36.6, 100, 100, 8.6, 0.366 | n.a. | 58.1, 48.6, 16.2, 100, 100, 43.0, 0.162 | 58.8, 60.5, 63.0, 54.5, 77.3, 37.5, 0.175 | 0.238 | 0.308 | 9, 0.004 |

Results shown as area under the curve [%], accuracy [%], sensitivity [%], specificity [%], positive predictive value [%], negative predictive value [%], Youden’s index. *Optimal cut-off in our previous study [15]. **Optimal cut-off within the developmental dataset [9]. ***Optimal cut-off determined for each study, shown with corresponding accuracy [%], sensitivity [%], specificity [%], positive predictive value [%], negative predictive value [%], and Youden’s index. ****Only Meena et al. [14] and Debaugnies et al. [19] considered. *****Statistical analyses performed using sign test. aHLH-2004, adjusted HLH-2004 criteria. HLH, Hemophagocytic Lymphohistiocytosis. n.a., not applicable. OHI, optimized HLH inflammatory. oHLH-2004, optimized HLH-2004 criteria. revHLH-2004, revised HLH-2004 criteria. shHLH-2004, shortened HLH-2004 criteria. sIL-2R, soluble interleukin-2 receptor.

**Supplement Table 6. Validation of original, modified and our newly developed HLH diagnostic criteria sets and biomarkers (Sensitivity analyses for patients of at least 6 obtained HLH-2004 criteria).**

|  | Fulfilled criteria or cut-off | Fardet et al., 2016 [9] | Debaugnies et al., 2016 [10] | Horrillo et al., 2019 [20] | Meena et al., 2020 [14] | Debaugnies et al., 2021 [19] | Lopez Marcos et al., 2021 [21] | Smits et al., 2021 [22] | Oh et al., 2021 [23] | Yao H et al., 2021 [24] | Yao S et al., 2021 [25] | Ammouri et al., 2022 [26]**** | He et al., 2022 [27] | Bilston et al., 2022 [12] | Youden’s index mean | Youden’s index mean (ICU cohorts)***** | Validation cohorts performing worse than respective HLH-2004 (cut-off 4) [n, p-value]****** |
| --- | --- | --- | --- | --- | --- | --- | --- | --- | --- | --- | --- | --- | --- | --- | --- | --- | --- |
| HLH-2004 [4] | 4* | 91.5, 85.8, 88.0, 81.9, 89.8, 79.1, 0.699 | 83.1, 70.9, 75.8, 67.4, 62.5, 79.5, 0.431 | 92.7, 87.2, 79.6, 78.8, 78.8, 97.6, 0.765 | 91.3, 75.6, 87.5, 74.4, 25.9, 98.3, 0.619 | 96.7, 91.3, 69.2, 94.9, 69.2, 94.9, 0.642 | 86.1, 66.7, 100, 25.0, 62.5, 100, 0.250 | 94.4, 89.4, 93.8, 84.5, 87.1, 92.5, 0.783 | 83.3, 75.2, 90.0, 53.7, 74.0, 78.6, 0.437 | 98.8, 95.0, 98.2, 93.3, 88.7, 99.0, 0.915 | 98.7, 95.6, 96.1, 90.9, 99.0, 71.4, 0.870 | - | 89.6, 79.5, 71.8, 91.3, 92.7, 67.7, 0.631 | 93.6, 88.6, 84.5, 89.3, 56.9, 97.2, 0.738 | 0.648 | 0.631 | - |
|  | 5 | 91.5, 68.2, 51.3, 98.8, 98.7, 52.9, 0.501 | 83.1, 77.2, 54.5, 93.5, 85.7, 74.1, 0.480 | 92.7, 78.7, 57.1, 96.2, 92.3, 73.5, 0.533 | 91.3, 91.9, 75.0, 93.6, 54.5, 97.3, 0.686 | 96.7, 92.4, 53.8, 98.7, 87.5, 92.9, 0.526 | 86.1, 77.8, 73.3, 83.3, 84.6, 71.4, 0.567 | 94.4, 87.0, 78.5, 96.6, 96.2, 80.0, 0.750 | 83.3, 79.2, 78.3, 80.5, 85.5, 71.7, 0.588 | 98.8, 91.9, 76.8, 100, 100, 89.0, 0.768 | 98.7, 84.2, 82.5, 100, 100, 37.9, 0.825 | - | 89.6, 62.4, 38.0, 100, 100, 51.1, 0.380 | 93.6, 92.4, 59.8, 97.9, 82.9, 93.5, 0.577 | 0.598 | 0.606 | 8, 0.388 |
| aHLH-2004 | 4 | 92.2, 83.7, 79.3, 91.6, 94.4, 71.0, 0.837 | 81.4, 72.2, 63.6, 78.3, 67.7, 75.0, 0.419 | 84.3, 78.7, 73.8, 82.7, 77.5, 79.6, 0.565 | 88.7, 86.0, 87.5, 85.9, 38.9, 98.5, 0.734 | 96.8, 92.4, 69.2, 96.2, 75.0, 95.0, 0.654 | 85.0, 70.4, 93.3, 41.7, 66.7, 83.3, 0.350 | 92.7, 87.8, 86.2, 89.7, 90.3, 85.2, 0.758 | 85.9, 80.2, 88.3, 68.3, 80.3, 80.0, 0.566 | 98.7, 95.7, 98.2, 94.3, 90.2, 99.0, 0.925 | 99.1, 90.4, 89.3, 100, 100, 50.0, 0.893 | - | 84.5, 69.2, 53.5, 93.5, 92.7, 56.6, 0.470 | 95.6, 92.0, 81.4, 93.8, 68.7, 96.8, 0.752 | 0.660 | 0.694 | 4, 0.388 |
| oHLH-2004 | 4 | 93.0, 76.4, 65.3, 96.4, 97.0, 60.6, 0.764 | 81.1, 73.4, 63.6, 80.4, 70.0, 75.5, 0.441 | 80.6, 71.3, 47.6, 90.4, 80.0, 68.1, 0.380 | 92.9, 90.7, 87.5, 91.0, 50.0, 98.6, 0.785 | 96.5, 91.3, 61.5, 96.2, 72.7, 93.8, 0.577 | 79.4, 70.4, 80.0, 58.3, 70.6, 70.0, 0.383 | 90.0, 83.7, 81.5, 86.2, 86.9, 80.6, 0.677 | 86.0, 83.2, 85.0, 80.5, 86.4, 78.6, 0.655 | 98.6, 95.0, 89.3, 98.1, 96.2, 94.5, 0.874 | 99.0, 86.8, 85.4, 100, 100, 42.3, 0.854 | - | 83.5, 65.8, 47.9, 93.5, 91.9, 53.8, 0.414 | 96.0, 92.6, 74.2, 95.7, 74.2, 95.7, 0.699 | 0.625 | 0.681 | 7, 0.774 |
| revHLH-2004 | 3 | 91.5, 80.7, 97.3, 50.6, 78.1, 91.3, 0.479 | 83.1, 65.8, 93.9, 45.7, 55.4, 91.3, 0.396 | 92.7, 52.1, 100, 13.5, 48.3, 100, 0.135 | 91.3, 48.8, 100, 43.6, 15.4, 100, 0.436 | 96.7, 89.1, 100, 87.3, 56.5, 100, 0.873 | 86.1, 63.0, 100, 16.7, 60.0, 100, 0.167 | 94.7, 77.2, 98.5, 53.4, 70.3, 96.9, 0.519 | 81.8, 70.3, 93.3, 36.6, 68.3, 78.9, 0.299 | 95.4, 69.6, 98.2, 54.3, 53.4, 98.3, 0.525 | 97.4, 95.6, 97.1, 81.8, 98.0, 75.0, 0.789 | - | 89.7, 81.2, 78.9, 84.8, 88.9, 72.2, 0.637 | 93.6, 72.7, 95.9, 68.8, 34.1, 99.0, 0.647 | 0.492 | 0.655 | 10, 0.039 |
|  | 4 | 91.5, 85.8, 88.0, 81.9, 89.8, 79.1, 0.699 | 83.1, 70.9, 75.8, 67.4, 62.5, 79.5, 0.431 | 92.7, 87.2, 97.6, 78.8, 78.8, 97.6, 0.765 | 91.3, 75.6, 87.5, 74.4, 25.9, 98.3, 0.619 | 96.7, 91.3, 69.2, 94.9, 69.2, 94.9, 0.642 | 86.1, 66.7, 100, 25.0, 62.5, 100, 0.250 | 94.7, 88.6, 90.8, 86.2, 88.1, 89.3, 0.770 | 81.8, 78.2, 83.3, 70.7, 80.6, 74.4, 0.541 | 95.4, 91.9, 89.3, 93.3, 87.7, 94.2, 0.826 | 97.4, 93.0, 93.2, 90.9, 99.0, 58.8, 0.841 | - | 89.7, 72.6, 57.7, 95.7, 95.3, 59.5, 0.534 | 93.6, 88.6, 84.5, 89.3, 56.9, 97.2, 0.738 | 0.638 | 0.631 | 4, 0.375 |
|  | 5 | 91.5, 68.2, 51.3, 98.8, 98.7, 52.9, 0.501 | 83.1, 77.2, 54.5, 93.5, 85.7, 74.1, 0.480 | 92.7, 78.7, 57.1, 96.2, 92.3, 73.5, 0.533 | 91.3, 91.9, 75.0, 93.6, 54.5, 97.3, 0.686 | 96.7, 92.4, 53.8, 98.7, 87.5, 92.9, 0.526 | 86.1, 77.8, 73.3, 83.3, 84.6, 71.4, 0.567 | 94.7, 84.6, 73.8, 96.6, 96.0, 76.7, 0.704 | 81.8, 71.3, 60.0, 87.8, 87.8, 60.0, 0.478 | 95.4, 86.3, 60.7, 100, 100, 82.7, 0.607 | 97.4, 75.4, 72.8, 100, 100, 28.2, 0.728 | - | 89.7, 56.4, 28.2, 100, 100, 47.4, 0.282 | 93.6, 92.4, 59.8, 97.9., 82.9, 93.5, 0.577 | 0.556 | 0.606 | 8, 0.388 |
| shHLH-2004 | 3 | 88.1, 81.1, 92.7, 60.2, 80.8, 82.0, 0.529 | 81.3, 67.1, 78.8, 58.7, 57.8, 79.4, 0.375 | 74.1, 58.5, 97.6, 26.9, 51.9, 93.3, 0.245 | 85.3, 55.8, 87.5, 52.6, 15.9, 97.6, 0.401 | 93.2, 84.8, 69.2, 87.3, 47.4, 94.5, 0.566 | 82.2, 63.0, 100, 16.7, 60.0, 100, 0.167 | 91.5, 76.4, 95.4, 55.2, 70.5, 91.4, 0.506 | 71.4, 66.3, 85.0, 39.0, 67.1, 64.0, 0.240 | 90.5, 68.9, 94.6, 55.2, 53.0, 95.1, 0.499 | 95.1, 94.7, 96.1, 81.8, 98.0, 69.2, 0.779 | - | 90.9, 83.8, 78.9, 91.3, 93.3, 73.7, 0.702 | 87.9, 73.0, 90.7, 70.0, 33.7, 97.8, 0.607 | 0.468 | 0.484 | 11, 0.006 |
|  | 4 | 88.1, 75.1, 65.3, 92.8, 94.2, 59.7, 0.581 | 81.3, 74.7, 66.7, 80.4, 71.0, 77.1, 0.471 | 74.1, 70.2, 57.1, 80.8, 70.6, 70.0, 0.379 | 85.3, 86.0, 75.0, 87.2, 37.5, 97.1, 0.622 | 93.2, 91.3, 69.2, 94.9, 69.2, 94.9, 0.642 | 82.2, 66.7, 100, 25.0, 62.5, 100, 0.250 | 91.5, 86.2, 86.2, 86.2, 87.5, 84.7, 0.724 | 71.4, 67.3, 65.0, 70.7, 76.5, 58.0, 0.357 | 90.5, 88.2, 75.0, 95.2, 89.4, 87.7, 0.702 | 95.1, 82.5, 81.6, 90.9, 98.8, 34.5, 0.725 | - | 90.9, 65.0, 43.7, 97.8, 96.9, 52.9, 0.415 | 87.9, 86.9, 64.9, 90.6, 53.8, 93.9, 0.556 | 0.535 | 0.632 | 8, 0.109 |
|  | 5 | 88.1, 52.8, 26.7, 100, 100, 43.0, 0.267 | 81.3, 74.7, 42.4, 97.8, 93.3, 70.3, 0.403 | 74.1, 59.6, 14.3, 96.2, 75.0, 58.1, 0.104 | 85.3, 91.9, 25.0, 98.7, 66.7, 92.8, 0.237 | 93.2, 93.5, 53.8, 100, 100, 92.9, 0.538 | 82.2, 74.1, 60.0, 91.7, 90.0, 64.7, 0.517 | 91.5, 75.6, 56.9, 96.6, 94.9, 66.7, 0.535 | 71.4, 51.5, 21.7, 95.1, 86.7, 45.3, 0.168 | 90.5, 78.9, 39.3, 100, 100, 75.5, 0.393 | 95.1, 54.4, 49.5, 100, 100, 17.5, 0.495 | - | 90.9, 52.1, 21.1, 100, 100, 45.1, 0.211 | 87.9, 87.4, 21.6, 98.4, 70.0, 88.2, 0.201 | 0.339 | 0.388 | 11, 0.006 |
| HScore [9] | 168* | 97.0, 92.3, 96.7, 84.3, 91.8, 93.3, 0.810 | 86.7, 77.2, 90.9, 67.4, 66.7, 91.2, 0.583 | 81.1, 70.2, 88.1, 55.8, 61.7, 85.3, 0.439 | 85.3, 76.7, 87.5, 75.6, 26.9, 98.3, 0.631 | 97.2, 94.6, 100, 93.7, 72.2, 100, 0.937 | 79.4, 59.3, 86.7, 25.0, 59.1, 60.0, 0.117 | 90.8, 76.4, 56.9, 98.3, 97.4, 67.1, 0.552 | 78.5, 72.3, 73.3, 70.7, 78.6, 64.4, 0.441 | 99.1, 90.1, 98.2, 85.7, 78.6, 98.9, 0.839 | 97.8, 81.6, 79.6, 100, 100, 34.4, 0.796 | - | 90.7, 77.8, 67.6, 93.5, 94.1, 65.2, 0.611 | 96.6, 91.2, 89.7, 91.5, 64.0, 98.1, 0.812 | 0.631 | 0.784 | 6, 1.000 |
|  | 169** | 97.0, 92.3, 96.7, 84.3, 91.8, 93.3, 0.810 | 86.7, 75.9, 87.9, 67.4, 65.9, 88.6, 0.553 | 81.1, 71.3, 88.1, 57.7, 62.7, 85.7, 0.458 | 85.3, 76.7, 87.5, 75.6, 26.9, 98.3, 0.631 | 97.2, 94.6, 100, 93.7, 72.2, 100, 0.937 | 79.4, 66.7, 86.7, 41.7, 65.0, 71.4, 0.283 | 90.8, 76.4, 56.9, 98.3, 97.4, 67.1, 0.552 | 78.5, 72.3, 73.3, 70.7, 78.6, 64.4, 0.441 | 99.1, 90.7, 98.2, 86.7, 79.7, 98.9, 0.849 | 97.8, 78.9, 76.7, 100, 100, 31.4, 0.767 | - | 90.7, 76.9, 66.2, 93.5, 94.0, 64.2, 0.597 | 96.6, 91.5, 89.7, 91.9, 64.9, 98.1, 0.815 | 0.641 | 0.784 | 5, 1.000 |
| Iteration 1 | 5 | 94.0, 68.2, 51.3, 98.8, 98.7, 52.9, 0.501 | 83.5, 77.2, 66.7, 84.8, 75.9, 78.0, 0.514 | 75.8, 60.6, 19.0, 94.2, 72.7, 59.0, 0.133 | 92.8, 91.9, 87.5, 92.3, 53.8, 98.6, 0.798 | 96.4, 90.2, 61.5, 94.9, 66.7, 93.8, 0.565 | 74.7, 63.0, 73.3, 50.0, 64.7, 60.0, 0.233 | 89.1, 83.7, 80.0, 87.9, 88.1, 79.7, 0.679 | 84.0, 79.2, 76.7, 82.9, 86.8, 70.8, 0.596 | 98.7, 90.7, 75.0, 99.0, 97.7, 88.1, 0.740 | 97.7, 71.1, 68.0, 100, 100, 25.0, 0.680 | - | 84.8, 58.1, 31.0, 100, 100, 48.4, 0.310 | 93.5, 91.1, 58.8, 96.5, 74.0, 93.3, 0.553 | 0.525 | 0.682 | 9, 0.146 |
|  | 6 | 93.5, 71.2, 56.0, 98.8, 98.8, 55.4, 0.548 | 82.5, 73.4, 63.6, 80.4, 70.0, 75.5, 0.441 | 73.6, 62.8, 31.0, 88.5, 68.4, 61.3, 0.194 | 92.0, 93.0, 75.0, 94.9, 60.0, 97.4, 0.699 | 96.4, 90.2, 53.8, 96.2, 70.0, 92.7, 0.500 | 78.6, 70.4, 80.0, 58.3, 70.6, 70.0, 0.383 | 89.4, 81.3, 72.3, 91.4, 90.4, 74.6, 0.637 | 83.9, 82.2, 83.3, 80.5, 86.2, 76.7, 0.638 | 99.5, 89.4, 69.6, 100, 100, 86.1, 0.696 | 98.2, 67.5, 64.1, 100, 100, 22.9, 0.641 | - | 86.4, 61.5, 36.6, 100, 100, 50.5, 0.366 | 93.8, 92.3, 61.9, 97.4, 80.0, 93.8, 0.593 | 0.528 | 0.600 | 8, 0.388 |
| Iteration 2 | 4 | 88.4, 53.6, 28.0, 100, 100, 43.5, 0.280 | 81.2, 78.5, 60.6, 91.3, 83.3, 76.4, 0.519 | 58.8, 55.3, 14.3, 88.5, 50.0, 56.1, 0.027 | 85.9, 89.5, 50.0, 93.6, 44.4, 94.8, 0.436 | 94.4, 93.5, 61.5, 98.7, 88.9, 94.0, 0.603 | 78.1, 77.8, 86.7, 66.7, 76.5, 80.0, 0.533 | 85.2, 72.4, 55.4, 91.4, 87.8, 64.6, 0.468 | 82.2, 73.3, 60.0, 92.7, 92.3, 61.3, 0.527 | 91.8, 85.7, 64.3, 97.1, 92.3, 83.6, 0.614 | 94.2, 57.9, 53.4, 100, 100, 18.6, 0.534 | - | 81.9, 55.6, 28.2, 97.8, 95.2, 46.9, 0.260 | 93.2, 90.4, 52.6, 96.7, 72.9, 92.4, 0.493 | 0.441 | 0.520 | 9, 0.146 |
| Iteration 5 | 6 | 90.0, 65.2, 46.7, 98.8, 98.6, 50.6, 0.455 | 83.1, 73.4, 60.6, 82.6, 71.4, 74.5, 0.432 | 65.0, 59.6, 31.0, 82.7, 59.1, 59.7, 0.136 | 82.9, 88.4, 37.5, 93.6, 37.5, 93.6, 0.311 | 96.3, 91.3, 61.5, 96.2, 72.7, 93.8, 0.577 | 77.5, 59.3, 80.0, 33.3, 60.0, 57.1, 0.133 | 90.0, 73.2, 53.8, 94.8, 92.1, 64.7, 0.487 | 72.8, 57.4, 41.7, 80.5, 75.8, 48.5, 0.222 | 97.9, 81.4, 46.4, 100, 100, 77.8, 0.464 | 90.6, 46.5, 40.8, 100, 100, 15.3, 0.408 | - | 85.7, 50.4, 18.3, 100, 100, 44.2, 0.183 | 91.1, 89.3, 50.5, 95.8, 67.1, 92.0, 0.464 | 0.356 | 0.444 | 11, 0.006 |
| Iteration 6 | 6 | 92.0, 67.0, 48.7, 100, 100, 51.9, 0.487 | 81.7, 73.4, 57.6, 84.8, 73.1, 73.6, 0.424 | 66.2, 60.6, 31.0, 84.6, 61.9, 60.3, 0.156 | 84.4, 89.5, 50.0, 93.6, 44.4, 94.8, 0.436 | 94.9, 92.4, 61.5, 97.5, 80.0, 93.9, 0.590 | 78.1, 70.4, 86.7, 50.0, 68.4, 75.0, 0.367 | 90.0, 69.9, 44.6, 98.3, 96.7, 61.3, 0.429 | 71.0, 54.5, 33.3, 85.4, 76.9, 46.7, 0.187 | 96.7, 78.9, 39.3, 100, 100, 75.5, 0.393 | 92.3, 42.1, 35.9, 100, 100, 14.3, 0.359 | - | 86.4, 47.9, 14.1, 100, 100, 43.0, 0.141 | 90.4, 88.6, 46.4, 95.7, 64.3, 91.4, 0.421 | 0.366 | 0.513 | 11, 0.006 |
| Iteration 8 | 6 | 88.4, 72.5, 63.3, 89.2, 91.3, 57.4, 0.525 | 83.8, 78.5, 69.7, 84.8, 76.7, 79.6, 0.545 | 61.4, 58.5, 50.0, 65.4, 53.8, 61.8, 0.154 | 89.3, 91.9, 75.0, 93.6, 54.5, 97.3, 0.686 | 95.6, 91.3, 61.5, 96.2, 72.7, 93.8, 0.577 | 62.2, 55.6, 93.3, 8.3, 56.0, 50.0, 0.017 | 87.0, 72.4, 53.8, 93.1, 89.7, 64.3, 0.469 | 72.7, 62.4, 55.0, 73.2, 75.0, 52.6, 0.282 | 98.1, 84.5, 55.4, 100, 100, 80.8, 0.554 | 89.3, 48.2, 42.7, 100, 100, 15.7, 0.427 | - | 85.0, 68.4, 52.1, 93.5, 92.5, 55.8, 0.456 | 91.2, 89.3, 57.7, 94.6, 64.4, 93.0, 0.524 | 0.435 | 0.632 | 10, 0.039 |
| Iteration 11 | 5 | 84.3, 56.2, 33.3, 97.6, 96.2, 44.8, 0.309 | 79.8, 77.2, 66.7, 84.8, 75.9, 78.0, 0.514 | 58.1, 55.3, 4.8, 96.2, 50.0, 55.6, 0.009 | 84.9, 90.7, 37.5, 96.2, 50.0, 93.8, 0.337 | 95.3, 91.3, 69.2, 94.9, 69.2, 94.9, 0.642 | 71.7, 63.0, 60.0, 66.7, 69.2, 57.1, 0.267 | 88.1, 69.1, 44.6, 96.6, 93.5, 60.9, 0.412 | 77.7, 68.3, 55.0, 87.8, 86.8, 57.1, 0.428 | 97.3, 85.1, 57.1, 100, 100, 81.4, 0.571 | 93.8, 64.0, 60.2, 100, 100, 21.2, 0.602 | - | 87.4, 66.7, 45.1, 100, 100, 54.1, 0.451 | 83.6, 87.1, 19.6, 98.4, 67.9, 87.9, 0.180 | 0.394 | 0.490 | 9, 0.065 |
| Iteration 12 | 5 | 86.9, 48.5, 20.0, 100, 100, 40.9, 0.200 | 83.1, 79.7, 57.6, 95.7, 90.5, 75.9, 0.532 | 68.0, 61.7, 28.6, 88.5, 66.7, 60.5, 0.170 | 88.9, 89.5, 37.5, 94.9, 42.9, 93.7, 0.324 | 97.0, 93.5, 61.5, 98.7, 88.9, 94.0, 0.603 | 69.7, 63.0, 66.7, 58.3, 66.7, 58.3, 0.250 | 85.2, 71.5, 55.4, 89.7, 85.7, 64.2, 0.450 | 77.5, 56.4, 31.7, 92.7, 86.4, 48.1, 0.243 | 94.3, 79.5, 41.1, 100, 100, 76.1, 0.411 | 90.6, 52.6, 47.6, 100, 100, 16.9, 0.476 | - | 82.3, 48.7, 15.5, 100, 100, 43.4, 0.155 | 89.9, 88.6, 33.0, 97.9, 72.7, 89.7, 0.309 | 0.344 | 0.464 | 10, 0.012 |
|  | 6 | 89.6, 56.7, 32.7, 100, 100, 45.1, 0.327 | 81.7, 73.4, 51.5, 89.1, 77.3, 71.9, 0.406 | 68.0, 61.7, 28.6, 88.5, 66.7, 60.5, 0.170 | 84.8, 91.9, 37.5, 97.4, 60.0, 93.8, 0.349 | 96.5, 92.4, 61.5, 97.5, 80.0, 93.9, 0.590 | 75.6, 59.3, 80.0, 33.3, 60.0, 57.1, 0.133 | 89.7, 80.5, 72.3, 89.7, 88.7, 74.3, 0.620 | 75.3, 61.4, 45.0, 85.4, 81.8, 51.5, 0.304 | 98.4, 85.1, 57.1, 100, 100, 81.4, 0.571 | 93.9, 55.3, 50.5, 100, 100, 17.7, 0.505 | - | 87.2, 57.3, 29.6, 100, 100, 47.9, 0.296 | 93.1, 90.5, 52.6, 96.9, 73.9, 92.4, 0.495 | 0.397 | 0.470 | 12, < 0.001 |
| Iteration 13 | 5 | 88.2, 52.4, 26.7, 98.8, 97.6, 42.7, 0.255 | 79.6, 81.0, 63.6, 93.5, 87.5, 78.2, 0.571 | 64.3, 61.7, 40.5, 78.8, 60.7, 62.1, 0.193 | 89.9, 91.9, 62.5, 94.9, 55.6, 96.1, 0.574 | 94.9, 90.2, 53.8, 96.2, 70.0, 92.7, 0.500 | 70.0, 63.0, 53.3, 75.0, 72.7, 56.3, 0.283 | 85.3, 73.2, 55.4, 93.1, 90.0, 65.1, 0.485 | 76.7, 59.4, 35.0, 95.1, 91.3, 50.0, 0.301 | 93.1, 75.8, 30.4, 100, 100, 72.9, 0.304 | 93.8, 48.2, 42.7, 100, 100, 15.7, 0.427 | - | 88.1, 51.3, 19.7, 100, 100, 44.7, 0.197 | 89.8, 88.0, 29.9, 97.7, 69.0, 89.2, 0.276 | 0.364 | 0.537 | 10, 0.012 |
|  | 6 | 92.0, 58.4, 35.3, 100, 100, 46.1, 0.353 | 80.0, 72.2, 48.5, 89.1, 76.2, 70.7, 0.376 | 62.0, 58.5, 28.6, 82.7, 57.1, 58.0, 0.113 | 86.5, 90.7, 37.5, 96.2, 50.0, 93.8, 0.337 | 95.3, 92.4, 61.5, 97.5, 80.0, 93.9, 0.590 | 77.8, 70.4, 86.7, 50.0, 68.4, 75.0, 0.367 | 89.5, 78.0, 64.6, 93.1, 91.3, 70.1, 0.577 | 73.9, 58.4, 36.7, 90.2, 84.6, 49.3, 0.269 | 97.3, 83.2, 51.8, 100, 100, 79.5, 0.518 | 95.4, 51.8, 46.6, 100, 100, 16.7, 0.466 | - | 89.0, 55.6, 26.8, 100, 100, 46.9, 0.268 | 92.3, 90.5, 51.5 97.1, 74.6, 92.3, 0.486 | 0.393 | 0.464 | 11, 0.006 |
|  | 7 | 91.4, 49.4, 21.3, 100, 100, 41.3, 0.213 | 82.3, 74.7, 54.5, 89.1, 78.3, 73.2, 0.437 | 63.8, 56.4, 9.5, 94.2, 57.1, 56.3, 0.038 | 82.4, 90.7, 25.0, 97.4, 50.0, 92.7, 0.224 | 95.7, 92.4, 53.8, 98.7, 87.5, 92.9, 0.526 | 81.7, 70.4, 73.3, 66.7, 73.3, 66.7, 0.400 | 89.1, 69.9, 47.7, 94.8, 91.2, 61.8, 0.425 | 75.5, 57.4, 31.7, 95.1, 90.5, 48.8, 0.268 | 97.4, 76.4, 32.1, 100, 100, 73.4, 0.321 | 94.3, 43.0, 36.9, 100, 100, 14.5, 0.369 | - | 87.6, 48.7, 15.5, 100, 100, 43.4, 0.155 | 92.3, 88.6, 30.9, 98.3, 75.0, 89.4, 0.292 | 0.306 | 0.375 | 10, 0.039 |
| Ferritin [µg/L] | 9083* | 86.9, 56.2, 34.0, 96.4, 94.4, 44.7, 0.304 | 73.8, 67.9, 42.4, 86.7, 70.0, 67.2, 0.291 | 66.9, 62.8, 23.8, 94.2, 76.9, 60.5, 0.180 | 70.7, 84.9, 50.0, 88.5, 30.8, 94.5, 0.385 | 92.5, 94.6, 69.2, 98.7, 90.0, 95.1, 0.680 | 78.3, 48.1, 6.7, 100, 100, 46.2, 0.067 | 71.4, 56.3, 28.1, 89.1, 75.0, 51.6, 0.172 | 73.7, 62.4, 48.3, 82.9, 80.6, 52.3, 0.313 | 80.1, 73.9, 64.3, 79.0, 62.1, 80.6, 0.433 | 90.0, 28.9, 21.4, 100, 100, 12.0, 0.214 | - | 88.7, 42.1, 4.3, 100, 100, 40.5, 0.043 | 88.6, 88.1, 59.8, 92.9, 58.6, 93.2, 0.527 | 0.301 | 0.533 | 11, 0.006 |
| sIL-2R [U/mL] | 4621* | n.a. | 71.1, 34.5, 5.0, 100, 100, 32.1, 0.050 | n.a. | n.a. | 87.4, 90.2, 30.8, 100, 100, 89.8, 0.308 | 61.1, 45.5, 33.3, 100, 100, 25.0, 0.333 | 80.0, 64.3, 37.9, 92.6, 84.6, 58.1, 0.305 | 72.0, 51.2, 37.5, 90.9, 92.3, 33.3, 0.284 | 89.6, 9.8, 4.2, 100, 100, 6.1, 0.042 | 90.4, 36.4, 32.7, 100, 100, 8.1, 0.327 | - | 75.0, 46.5, 14.3, 97.7, 90.9, 41.7, 0.120 | 58.2, 57.9, 55.6, 63.6, 78.9, 36.8, 0.192 | 0.218 | 0.308 | 8, 0.039 |
| OHI index [11] | sIL-2R > 3900 U/mL and ferritin > 1000 µg/L | n.a. | 52.5, 32.1, 5.0, 100, 100, 29.6, 0.050 | n.a. | n.a. | 65.4, 30.8, 30.8, 100, 100, 89.7, 0.308 | 66.7, 45.5, 33.3, 100, 100, 25.0, 0.333 | 66.2, 64.9, 36.2, 96.2, 91.3, 58.0, 0.324 | 65.8, 53.5, 40.6, 90.9, 92.9, 34.5, 0.315 | 55.2, 15.7, 10.4, 100, 100, 6.5, 0.104 | 68.3, 40.2, 36.6, 100, 100, 8.6, 0.366 | - | 58.1, 48.6, 16.2, 100, 100, 43.0, 0.162 | 58.8, 60.5, 63.0, 54.5, 77.3, 37.5, 0.175 | 0.237 | 0.308 | 8, 0.039 |

Results shown as area under the curve [%], accuracy [%], sensitivity [%], specificity [%], positive predictive value [%], negative predictive value [%], Youden’s index. *Optimal cut-off in our previous study [15]. **Optimal cut-off within the developmental dataset [9]. ***Optimal cut-off determined for each study, shown with corresponding accuracy [%], sensitivity [%], specificity [%], positive predictive value [%], negative predictive value [%], and Youden’s index. ****only HLH patients in this cohort. *****Only Meena et al. [14] and Debaugnies et al. [19] considered. ******Statistical analyses performed using sign test. aHLH-2004, adjusted HLH-2004 criteria. HLH, Hemophagocytic Lymphohistiocytosis. n.a., not applicable. OHI, optimized HLH inflammatory. oHLH-2004, optimized HLH-2004 criteria. revHLH-2004, revised HLH-2004 criteria. shHLH-2004, shortened HLH-2004 criteria. sIL-2R, soluble interleukin-2 receptor.

**Supplement Table 7. Validation of original, modified and our newly developed HLH diagnostic criteria sets and biomarkers (Sensitivity analyses for cohorts with HLH diagnosis based on expert review).**

|  | Fulfilled criteria or cut-off | Fardet et al., 2016 [9] | Debaugnies et al., 2016 [10] | Meena et al., 2020 [14] | Debaugnies et al., 2021 [19] | Yao H et al., 2021 [24] | Bilston et al., 2022 [12] | Youden’s index mean | Youden’s index mean (ICU cohorts)**** | Validation cohorts performing worse than respective HLH-2004 (cut-off 4) [n, p-value]***** |
| --- | --- | --- | --- | --- | --- | --- | --- | --- | --- | --- |
| HLH-2004 [4] | 4* | 90.6, 84.6, 85.8, 82.7, 88.5, 78.9, 0.685 | 89.7, 81.3, 77.1, 82.7, 60.0, 91.5, 0.598 | 97.6, 93.9, 90.0, 94.0, 25.7, 99.8, 0.840 | 94.3, 92.5, 64.3, 96.2, 69.2, 95.3, 0.605 | 98.9, 95.2, 98.2, 93.8, 88.7, 99.1, 0.920 | 94.6, 91.0, 83.7, 91.9, 55.8, 97.9, 0.755 | 0.734 | 0.723 | - |
|  | 5 | 90.6, 67.7, 48.1, 98.1, 97.5, 54.8, 0.462 | 89.7, 86.3, 54.3, 97.1, 86.4, 86.3, 0.514 | 97.6, 98.0, 60.0, 98.9, 54.5, 99.1, 0.588 | 94.3, 93.3, 50.0, 99.1, 87.5, 93.8, 0.491 | 98.9, 92.3, 76.8, 100, 100, 89.6, 0.768 | 94.6, 94.2, 59.2, 98.5, 82.9, 95.2, 0.577 | 0.567 | 0.540 | 6, 0.031 |
| aHLH-2004 | 4 | 91.2, 82.7, 77.8, 90.4, 92.6, 72.3, 0.682 | 87.5, 82.7, 65.7, 88.5, 65.7, 88.5, 0.542 | 96.6, 96.4, 80,0, 96.8, 36.4, 99.5, 0.768 | 95.6, 93.3, 64.3, 97.2, 75.0, 95.4, 0.615 | 98.8, 95.8, 98.2, 94.6, 90.2, 99.1, 0.929 | 96.4, 93.8, 80.6, 95.4, 68.1, 97.6, 0.760 | 0.716 | 0.692 | 3, 1.000 |
| oHLH-2004 | 4 | 92.3, 76.7, 63.6, 97.1, 97.2, 63.1, 0.607 | 87.4, 83.5, 65.7, 89.4, 67.6, 88.6, 0.551 | 97.7, 97.8, 70.0, 98.4, 50.0, 99.3, 0.684 | 95.5, 92.5, 57.1, 97.2, 72.7, 94.5, 0.543 | 98.6, 95.2, 89.3, 98.2, 96.2, 94.8, 0.875 | 96.7, 94.2, 73.5, 96.7, 73.5, 96.7, 0.702 | 0.660 | 0.614 | 6, 0.031 |
| revHLH-2004 | 3 | 90.6, 80.1, 96.3, 54.8, 76.8, 90.5, 0.511 | 89.7, 69.8, 94.3, 61.5, 45.2, 97.0, 0.558 | 97.6, 83.6, 100, 83.2, 12.0, 100, 0.832 | 94.3, 90.0, 92.9, 89.6, 54.2, 99.0, 0.825 | 95.5, 68.5, 94.6, 55.4, 51.5, 95.4, 0.500 | 94.5, 77.1, 94.9, 75.0, 31.7, 99.2, 0.699 | 0.654 | 0.829 | 5, 0.219 |
|  | 4 | 90.6, 84.6, 85.8, 82.7, 88.5, 78.9, 0.685 | 89.7, 81.3, 77.1, 82.7, 60.0, 91.5, 0.598 | 97.6, 93.9, 90.0, 94.0, 25.7, 99.8, 0.840 | 94.3, 92.5, 64.3, 96.2, 69.2, 95.3, 0.605 | 95.5, 92.3, 89.3, 93.8, 87.7, 94.6, 0.830 | 94.5, 91.0, 83.7, 91.9, 55.8, 97.9, 0.755 | 0.719 | 0.723 | 1, 1.000 |
|  | 5 | 90.6, 67.7, 48.1, 98.1, 97.5, 54.8, 0.462 | 89.7, 86.3, 54.3, 97.1, 86.4, 86.3, 0.514 | 97.6, 98.0, 60.0, 98.9, 54.5, 99.1, 0.589 | 94.3, 93.3, 50.0, 99.1, 87.5, 93.8, 0.491 | 95.5, 86.9, 60.7, 100, 100, 83.6, 0.607 | 94.5, 94.2, 59.2, 98.5, 82.9, 95.2, 0.577 | 0.540 | 0.540 | 6, 0.031 |
| shHLH-2004 | 3 | 87.3, 80.1, 90.1, 64.4, 79.8, 80.7. 0.545 | 89.0, 76.3, 80.0, 75.0, 51.9, 91.8, 0.550 | 94.7, 84.9, 90.0, 84.8, 12.0, 99.7, 0.748 | 91.3, 86.7, 64.3, 89.6, 45.0, 95.0, 0.539 | 90.6, 95.2, 98.2, 93.8, 88.7, 99.1, 0.920 | 89.6, 77.4, 89.8, 75.8, 31.3, 98.4, 0.656 | 0.660 | 0.644 | 5, 0.063 |
|  | 4 | 87.3, 73.7, 61.1, 93.3, 93.4, 60.6, 0.544 | 89.0, 84.9, 65.7, 91.3, 71.9, 88.8, 0.571 | 94.7, 95.7, 70.0, 96.3, 30.4, 99.3, 0.663 | 91.3, 92.5, 64.3, 96.2, 69.2, 95.3, 0.605 | 90.6, 88.7, 75.0, 95.5, 89.4, 88.4, 0.705 | 89.6, 89.7, 64.3, 92.9, 52.5, 95.5, 0.572 | 0.610 | 0.634 | 5, 0.063 |
|  | 5 | 87.3, 54.1, 24.7, 100, 100, 46.0, 0.247 | 89.0, 84.2, 40.0, 99.0, 93.3, 83.1, 0.390 | 94.7, 98.0, 20.0, 99.8, 66.7, 98.2, 0.198 | 91.3, 94.2, 50.0, 100, 100, 93.8, 0.500 | 90.6, 79.8, 39.3, 100, 100, 76.7, 0.393 | 89.6, 90.4, 21.4, 98.9, 70.0, 91.1, 0.203 | 0.322 | 0.349 | 6, 0.031 |
| HScore [9] | 168* | 96.9, 92.1, 96.3, 85.6, 91.2, 93.7, 0.819 | 92.7, 84.2, 91.4, 81.7, 62.7, 96.6, 0.732 | 95.6, 93.7, 80.0, 94.0, 23.5, 99.5, 0.740 | 97.3, 95.0, 92.9, 95.3, 72.2, 99.0, 0.881 | 99.0, 89.9, 98.2, 85.7, 77.5, 99.0, 0.839 | 97.1, 93.3, 88.8, 93.9, 64.0, 98.6, 0.826 | 0.806 | 0.811 | 2, 0.687 |
|  | 169** | 96.9, 92.1, 96.3, 85.6, 91.2, 93.7, 0.819 | 92.7, 83.5, 88.6, 81.7, 62.0, 95.5, 0.703 | 95.6, 93.7, 80.0, 94.0, 23.5, 99.5, 0.740 | 97.3, 95.0, 92.9, 95.3, 72.2, 99.0, 0.881 | 99.0, 90.5, 98.2, 86.6, 78.6, 99.0, 0.848 | 97.1, 93.5, 88.8, 94.1, 64.9, 98.6, 0.829 | 0.803 | 0.811 | 2, 0.687 |
|  | optimal*** | 177; 96.9, 91.4, 92.0, 90.4, 93.7, 87.9, 0.824 | 168; 92.7, 84.2, 91.4, 81.7, 62.7, 96.6, 0.732 | 167; 95.6, 93.7, 90.0, 93.8, 25.0, 99.8, 0.838 | 120; 97.3, 90.8, 100, 89.6, 56.0, 100, 0.896 | 173; 99.0, 94.6, 98.2, 92.9, 87.3, 99.0, 0.911 | 157; 97.1, 92.3, 94.9, 92.0, 59.2, 99.3, 0.869 | - | - | - |
| Iteration 1 | 5 | 92.3, 69.2, 50.0, 99.0, 98.8, 56.0, 0.490 | 90.3, 87.1, 68.6, 93.3, 77.4, 89.8, 0.618 | 97.4, 98.0, 70.0, 98.6, 53.8, 99.3, 0.686 | 96.4, 91.7, 57.1, 96.2, 66.7, 94.4, 0.534 | 98.8, 91.1, 75.0, 99.1, 97.7, 88.8, 0.741 | 94.7, 58.2, 93.2,  97.5, 74.0, 95.0, 0.557 | 0.604 | 0.610 | 5, 0.219 |
|  | 6 | 92.5, 72.2, 54.9, 99.0, 98.9, 58.5, 0.540 | 89.1, 84.9, 65.7, 91.3, 71.9, 88.8, 0.571 | 96.8, 98.0, 60.0, 98.9, 54.5, 99.1, 0.589 | 96.5, 91.7, 50.0, 97.2, 70.0, 93.6, 0.472 | 99.5, 89.9, 69.6, 100, 100, 86.8, 0.696 | 94.7, 94.0, 61.2, 98.0, 78.9, 95.4, 0.592 | 0.577 | 0.437 | 6, 0.031 |
| Iteration 2 | 4 | 87.9, 55.3, 26.5, 100, 100, 46.6, 0.265 | 88.3, 86.3, 60.0, 95.2, 80.8, 87.6, 0.552 | 95.0, 97.8, 50.0, 98.9, 50.0, 98.9, 0.489 | 93.5, 94.2, 57.1, 99.1, 88.9, 94.6, 0.562 | 91.3, 60.7, 64.3, 96.4, 90.0, 84.4, 0.607 | 94.4, 92.6, 52.0, 97.6, 72.9, 94.3, 0.497 | 0.495 | 0.526 | 6, 0.031 |
| Iteration 5 | 6 | 90.4, 65.8, 44.4, 99.0, 98.6, 53.4, 0.435 | 89.5, 82.7, 60.0, 90.4, 67.7, 87.0, 0.504 | 94.3, 97.3, 30.0, 98.9, 37.5, 98.4, 0.289 | 96.2, 92.5, 57.1, 97.2, 72.7, 94.5, 0.543 | 97.7, 82.1, 46.4, 100, 100, 78.9, 0.464 | 92.5, 91.9, 50.0, 97.0, 67.1, 94.1, 0.470 | 0.451 | 0.416 | 6, 0.031 |
| Iteration 6 | 6 | 91.6, 66.5, 45.1, 100, 100, 53.9, 0.451 | 89.1, 82.7, 57.1, 91.3, 69.0, 86.4, 0.485 | 94.2, 97.1, 40.0, 98.4, 36.4, 98.6, 0.384 | 95.1, 93.3, 57.1, 98.1, 80.0, 94.5, 0.553 | 96.6, 79.8, 39.3, 100, 100, 76.7, 0.393 | 91.7, 91.2, 45.9, 96.7, 63.4, 93.6, 0.427 | 0.449 | 0.469 | 6, 0.031 |
| Iteration 8 | 6 | 89.2, 74.1, 63.6, 90.4, 91.2, 61.4, 0.540 | 89.8, 84.9, 68.6, 90.4, 70.6, 89.5, 0.590 | 95.5, 97.5, 60.0, 98.4, 46.2, 99.1, 0.584 | 95.7, 92.5, 57.1, 97.2, 72.7, 94.5, 0.543 | 98.2, 85.1, 55.4, 100, 100, 81.8, 0.554 | 92.7, 91.9, 57.1, 96.1, 64.4, 94.8, 0.533 | 0.557 | 0.564 | 6, 0.031 |
| Iteration 11 | 5 | 83.6, 58.6, 33.3, 98.1, 96.4, 48.6, 0.314 | 86.0, 84.9, 65.7, 91.3, 71.9, 88.8, 0.571 | 95.4, 98.0, 40.0, 99.3, 57.1, 98.6, 0.393 | 93.4, 92.5, 64.3, 96.2, 69.2, 95.3, 0.605 | 97.3, 85.7, 57.1, 100, 100, 82.4, 0.571 | 86.2, 90.2, 19.4, 98.9, 67.9, 90.9, 0.183 | 0.440 | 0.499 | 5, 0.063 |
| Iteration 12 | 5 | 85.6, 50.8, 19.1, 100, 100, 44.3, 0.191 | 90.1, 87.1, 54.3, 98.1, 90.5, 86.4, 0.524 | 96.5, 97.5, 30.0, 99.1, 42.9, 98.4, 0.291 | 96.6, 94.2, 57.1, 99.1, 88.9, 94.6, 0.562 | 94.2, 80.4, 41.1, 80.4, 100, 77.2, 0.411 | 91.8, 91.3, 32.7, 98.5, 72.7, 92.3, 0.312 | 0.382 | 0.427 | 6, 0.031 |
|  | 6 | 90.4, 58.3, 31.5, 100, 100, 48.4, 0.315 | 88.5, 83.5, 51.4, 94.2, 75.0, 85.2, 0.457 | 94.4, 98.0, 30.0, 99.5, 60.0, 98.4, 0.295 | 96.6, 93.3, 57.1, 98.1, 80.0, 94.5, 0.553 | 98.2, 85.7, 57.1, 100, 100, 82.4, 0.571 | 94.1, 92.8, 52.0, 97.7, 73.9, 94.3, 0.498 | 0.448 | 0.424 | 6, 0.031 |
| Iteration 13 | 5 | 86.7, 53.8, 24.7, 99.0, 97.6, 45.8, 0.237 | 87.4, 87.8, 62.9, 96.2, 84.6, 88.5, 0.590 | 96.2, 97.5, 50.0, 98.6, 45.5, 98.8, 0.486 | 95.2, 91.7, 50.0, 97.2, 70.0, 93.6, 0.472 | 93.0, 76.8, 30.4, 100, 100, 74.2, 0.304 | 91.3, 90.7, 29.6, 98.2, 67.4, 91.9, 0.278 | 0.395 | 0.479 | 6, 0.031 |
|  | 6 | 91.9, 59.0, 32.7, 100, 100, 48.8, 0.327 | 87.9, 82.0, 48.6, 93.3, 70.8, 84.3, 0.418 | 94.4, 97.3, 30.0, 98.9, 37.5, 98.4, 0.289 | 95.7, 93.3, 57.1, 98.1, 80.0, 94.5, 0.553 | 97.2, 83.9, 51.8, 100, 100, 80.6, 0.518 | 93.3, 92.8, 51.0, 97.9, 74.6, 94.2, 0.489 | 0.432 | 0.421 | 6, 0.031 |
|  | 7 | 91.6, 51.1, 19.8, 100, 100, 44.4, 0.198 | 89.0, 84.2, 54.3, 94.2, 76.0, 86.0, 0.485 | 93.1, 97.8, 20.0, 99.5, 50.0, 98.2, 0.195 | 95.7, 93.3, 50.0, 99.1, 87.5, 93.8, 0.491 | 97.1, 77.4, 32.1, 100, 100, 74.7, 0.321 | 93.4, 91.3, 30.6, 98.7, 75.0, 92.1, 0.294 | 0.331 | 0.343 | 6, 0.031 |
| Ferritin [µg/L] | 9083* | 86.5, 56.8, 34.0, 96.6, 94.5, 45.7, 0.306 | 77.4, 73.7, 42.9, 90.6, 71.4, 74.4, 0.335 | 86.1, 95.1, 50.0, 96.1, 22.7, 98.8, 0.461 | 94.1, 95.8, 71.4, 99.1, 90.9, 96.3, 0.705 | 80.0, 74.4, 64.3, 79.5, 61.0, 81.7, 0.438 | 90.3, 91.0, 59.8, 94.7, 58.0, 95.1, 0.545 | 0.465 | 0.583 | 5, 0.219 |
|  | optimal*** | 1985; 86.5, 83.4, 86.9, 77.3, 86.9, 77.3, 0.642 | 3353; 77.4, 71.7, 68.6, 73.4, 58.5, 81.0, 0.420 | 1197; 86.1, 70.6, 90.0, 70.1, 6.5, 99.7, 0.601 | 1799; 94.1, 82.5, 92.9, 81.1, 39.4, 98.9, 0.740 | 9574; 80.0, 76.2, 64.3, 82.1, 64.3, 82.1, 0.464 | 6843; 90.3, 89.5, 73.2, 91.5, 51.1, 96.6, 0.647 | - | - | - |
| sIL-2R [U/mL] | 4621* | n.a. | 70.4, 40.6, 5.0, 100, 100, 38.7, 0.050 | n.a. | 83.9, 91.7, 28.6, 100, 100, 91.4, 0.286 | 89.6, 9.8, 4.2, 100, 100, 6.1, 0.042 | 58.2, 57.9, 55.6, 63.6, 78.9, 36.8, 0.192 | 0.143 | 0.286 | 4, 0.125 |
|  | optimal*** | n.a. | 462; 70.4, 65.6, 60.0, 75.0, 80.0, 52.9, 0.350 | n.a. | 576; 83.9, 84.2, 78.6, 84.9, 40.7, 96.8, 0.635 | 847; 89.6, 82.4, 81.3, 100, 100, 25.0, 0.813 | 4285; 58.2, 60.5, 59.3, 63.6, 80.0, 38.9, 0.229 | - | - | - |
| OHI index [11] | sIL-2R > 3900 U/mL and ferritin > 1000 µg/L | n.a. | 52.5, 32.1, 5.0, 100, 100, 29.6, 0.050 | n.a. | 64.3, 91.7, 28.6, 100, 100, 91.4, 0.286 | 55.2, 15.7, 10.4, 100, 100, 6.5, 0.104 | 58.8, 60.5, 63.0, 54.5, 77.3, 37.5, 0.175 | 0.154 | 0.286 | 4, 0.125 |

Results shown as area under the curve [%], accuracy [%], sensitivity [%], specificity [%], positive predictive value [%], negative predictive value [%], Youden’s index. *Optimal cut-off in our previous study [15]. **Optimal cut-off within the developmental dataset [9]. ***Optimal cut-off determined for each study, shown with corresponding accuracy [%], sensitivity [%], specificity [%], positive predictive value [%], negative predictive value [%], and Youden’s index. ****Only Meena et al. [14] and Debaugnies et al. [19] considered. *****Statistical analyses performed using sign test. aHLH-2004, adjusted HLH-2004 criteria. HLH, Hemophagocytic Lymphohistiocytosis. n.a., not applicable. OHI, optimized HLH inflammatory. oHLH-2004, optimized HLH-2004 criteria. revHLH-2004, revised HLH-2004 criteria. shHLH-2004, shortened HLH-2004 criteria. sIL-2R, soluble interleukin-2 receptor.

**Supplement Table 8. Best cut-offs and quality criteria of HLH diagnostic criteria sets within our dataset.**

|  | Ferritin [µg/L] (≥) | Fever [°C] (≥) | Splenomegaly | Hepatomegaly | Hemoglobin [g/dL]* (<) | Thrombocytes [/nL]* (<) | Leukocytes [/nL]* (<) | Hypertriglyceridemia [mg/dL]* (>) | Hypofibrinogenemia [g/L]* (<) | Hemophagocytosis | Reduced NK cell activity | sIL-2R [U/mL] (≥) | ASAT [U/L] (>) | Fulfilled criteria | Area under the curve [%] | Accuracy [%] | Sensitivity [%] | Specificity [%] | Positive predictive value [%] | Negative predictive value [%] | Youden’s index |
| --- | --- | --- | --- | --- | --- | --- | --- | --- | --- | --- | --- | --- | --- | --- | --- | --- | --- | --- | --- | --- | --- |
| oHLH-2004 | 3102 | 38.2 | yes | - | 7.8 81 0.08 | | | 332 1.5 | | yes | yes | 1706 | - | 4 | 99.5 | 97.8 | 100 | 97.8 | 40.8 | 100 | 0.9775 |
| Iteration 1** | 3102 | 38.2 | yes | - | 8.2 | 58 | 0.08 | 332 1.5 | | yes | yes | 1706 | - | 5 | 99.6 | 98.0 | 100 | 98.0 | 43.5 | 100 | 0.9799 |
| Iteration 1** | 3102 | 38.2 | yes | - | 8.2 | 81 | 8.5 | 332 1.5 | | yes | yes | 1706 | - | 6 | 99.7 | 98.6 | 100 | 98.6 | 52.6 | 100 | 0.9861 |
| Iteration 2 | 3102 | 38.2 | yes | - | 7.8 58 0.08 | | | 777 3.2 | | - | - | 1910 | - | 4 | 99.4 | 97.8 | 100 | 97.7 | 40.4 | 100 | 0.9772 |
| Iteration 3 | *** | *** | *** | *** | *** | | | *** | | *** | *** | *** | *** | *** | *** | *** | *** | *** | *** | *** | *** |
| Iteration 4 | 11456 | 38.2 | yes | - | 8.2 | 120 | 5.7 | 666 3.2 | | - | - | - | - | 5 | 99.3 | 97.0 | 100 | 96.9 | 33.6 | 100 | 0.9694 |
| Iteration 5 | 8323 | 38.2 | yes | - | 8.2 | 120 | 8.5 | 221 | 3.2 | - | - | - | - | 6 | 99.6 | 98.4 | 100 | 98.3 | 48.2 | 100 | 0.9834 |
| Iteration 6 | 3102 | 38.2 | yes | - | 8.2 | 120 | 7.6 | 221 | 1.5 | - | - | - | 2525 | 6 | 99.6 | 98.4 | 100 | 98.3 | 48.8 | 100 | 0.9837 |
| Iteration 7 | 8323 | 38.2 | yes | yes | 8.2 | 120 | 8.5 | 221 | 2.8 | - | - | - | - | 6 | 99.5 | 97.4 | 100 | 97.4 | 37.0 | 100 | 0.9737 |
| Iteration 8 | 8323 | 38.2 | yes | yes | 7.2 | 120 | 5.7 | 110 | 2.8 | - | - | - | 2525 | 6 | 99.5 | 97.7 | 100 | 97.6 | 39.6 | 100 | 0.9764 |
| Iteration 9 | *** | *** | *** | *** | *** | | | *** | | *** | *** | *** | *** | *** | *** | *** | *** | *** | *** | *** | *** |
| Iteration 10 | 3102 | 38.2 | yes | - | 8 120 0.08 | | | 221 1.5 | | - | - | - | 2525 | 4 | 99.1 | 97.1 | 100 | 97.0 | 34.2 | 100 | 0.9702 |
| Iteration 11 | 12500 | 38.4 | yes | - | 8.0 | 120 | 2.9 | 666 3.2 | | - | - | 1910 | - | 5 | 99.6 | 98.0 | 100 | 98.0 | 43.5 | 100 | 0.9799 |
| Iteration 12** | 8323 | 38.2 | yes | - | 8.2 | 58 | 0.08 | 554 | 3.2 | - | - | 1910 | - | 5 | 99.6 | 98.2 | 100 | 98.1 | 45.5 | 100 | 0.9814 |
| Iteration 12** | 8323 | 38.2 | yes | - | 7.0 | 120 | 8.5 | 221 | 3.2 | - | - | 1910 | - | 6 | 99.8 | 98.9 | 100 | 98.9 | 58.0 | 100 | 0.9888 |
| Iteration 13** | 3102 | 38.2 | yes | - | 8.0 | 81 | 0.08 | 554 | 1.5 | - | - | 1910 | 2525 | 5 | 99.5 | 98.2 | 100 | 98.2 | 46.5 | 100 | 0.9822 |
| Iteration 13** | 3102 | 38.2 | yes | - | 7.0 | 120 | 8.5 | 221 | 1.5 | - | - | 1910 | 1695 | 6 | 99.7 | 98.9 | 100 | 98.9 | 58.8 | 100 | 0.9892 |
| Iteration 13** | 3102 | 38.2 | yes | - | 8.0 | 104 | 8.5 | 221 | 3.2 | - | - | 1910 | 2525 | 7 | 99.8 | 99.4 | 100 | 99.3 | 70.2 | 100 | 0.9934 |

*Separate criteria when dividing line in cells, otherwise combined as in HLH-2004 criteria. **Same iteration shown for different numbers of fulfilled criteria as each Youden’s index ≥ 0.975. ***No results for Youden’s index ≥ 0.95. ASAT, aspartate aminotransferase. NK, natural killer. sIL-2R, soluble interleukin-2 receptor.

**Supplement Table 9. Detailed description of requested data and missing data rate of the validation cohorts.**

|  | Ferritin [µg/L] | Core body temperature [°C] | Splenomegaly | Hepatomegaly | Hemoglobin [g/dL] | Thrombocytes [/nL] | Leukocytes [/nL] | Triglycerides [mg/dL] | Fibrinogen [g/L] | Hemophagocytosis | Reduced NK cell activity | sIL-2R [U/mL] | ASAT [U/L] | Positive OHI index | Known underlying immunosuppression | At least 5 obtained HLH-2004 criteria | At least 6 obtained HLH-2004 criteria |
| --- | --- | --- | --- | --- | --- | --- | --- | --- | --- | --- | --- | --- | --- | --- | --- | --- | --- |
| Fardet et al., 2016 [9] | 2947 (1117 – 8368)  [9.4 %] | 39.0 (38.5 – 40.0)  [1.5 %] | 59.5 %  [1.5 %] | 64.3 %  [1.1 %] | 8.7 (7.6 – 9.9)  [0 %] | 68 (35 – 109)  [0 %] | 3.5 (1.9 – 7.7)  [0 %] | 233 (154 – 337)  [7.1 %] | 4.1 (2.6 – 5.8)  [2.6 %] | 62.8 %  [0 %] | -  [100 %] | -  [100 %] | 54 (29 – 134)  [0,4 %] | -  [100 %] | 40.2 %  [0 %] | 99.2 % | 87.6 % |
| Debaugnies et al., 2016 [10] | 2289 (558 – 7665)  [28.8 %] | 38.5 (37.0 – 39.0)  [35.3 %] | 29.0 %  [0.7 %] | 28.8 %  [0 %] | 7.9 (6.9 – 10.4)  [0 %] | 66 (14 – 175)  [0 %] | 2.4 (0.7 – 5.8)  [0 %] | 204 (132 – 350)  [42.4 %] | 2.6 (1.3 – 4.0)  [19.4 %] | 70.5 %  [0 %] | -  [100 %] | 444 (250 – 978)  [77.0 %] | 60 (30 – 165)  [4.3 %] | 3.6 % [79.9 %] | 49.6 %  [0 %] | 74.1 % | 56.8 % |
| Horrillo et al., 2019 [20] | 1969 (838 – 5784)  [5.4 %] | 38.3 (38.3 – 38.3)  [0 %] | 93.4 %  [4.5 %] | 29.2 %  [4.5 %] | 10.1 (8.9 – 11.2)  [0 %] | 91 (64 – 115)  [0 %] | 2.7 (2.0 – 3.3)  [0 %] | 196 (155 – 249)  [2.7 %] | 3.7 (2.8 – 4.7)  [7.2 %] | 50.5 %  [7.2 %] | -  [100 %] | -  [100 %] | 77 (36 – 162)  [1.8 %] | -  [100 %] | 25.2 %  [0 %] | 98.2 % | 84.7 % |
| Meena et al., 2020 [14] | 523 (191 – 1442)  [0 %] | 37.3 (36.9 – 37.9)  [0 %] | 10.3 %  [0 %] | 13.0 %  [0 %] | 8.9 (8.0 – 10.0)  [0 %] | 145 (69 – 276)  [0 %] | 8.9 (5.0 – 12.6)  [0 %] | 164 (90 – 297)  [54.2 %] | 3.4 (1.7 – 5.1)  [48.1 %] | 42.3 %  [78.2 %] | -  [100 %] | -  [100 %] | 53 (29 – 132)  [69.7 %] | -  [100 %] | 23.1 %  [0 %] | 70.8 % | 19.3 % |
| Debaugnies et al., 2021 [19] | 637 (239 - 2137)  [0 %] | 38.0 (37.0 – 38.7)  [17.5 %] | 13.9 %  [10.0 %] | 14.8 %  [10.0 %] | 10.0 (8.2 – 12.2)  [2.5 %] | 156 (51 – 251)  [2.5 %] | 11.0 (4.7 – 18.6)  [2.5 %] | 119 (78 – 200)  [19.2 %] | 5.6 (3.5 – 7.5)  [21.7 %] | 41.7 %  [80.0 %] | -  [100 %] | 293 (151 – 550)  [0 %] | 38 (20 – 98)  [3.3 %] | 3.3 % [0 %] | 19.2 %  [0 %] | 92.5 % | 76.7 % |
| Lopez Marcos et al., 2021 [21] | 801 (348 – 2739)  [31.5 %] | 38.3 (38.3 – 38.3)  [3.9 %] | 96.8 %  [1.6 %] | 73.4 %  [2.4 %] | 6.9 (6.1 – 8.1)  [9.4 %] | 66 (39 – 95)  [12.6 %] | 3.4 (2.3 – 5.0)  [13.4 %] | 275 (207 – 386)  [26.8 %] | 2.4 (1.5 – 3.0)  [55.1 %] | 33.3 %  [69.3 %] | -  [100 %] | 2280 (1440 – 14125)  [91.3 %] | 117 (56 – 241)  [19.7 %] | 27.3 %  [91.3 %] | 2.4 %  [0 %] | 61.4 % | 21.3 % |
| Smits et al., 2021 [22] | 1965 (657 – 6676)  [10.1 %] | 38.3 (37.3 – 38.3)  [12.1 %] | 47.7 % [0 %] | -  [100 %] | 5.8 (4.8 – 6.9)  [1.3 %] | 82 (32 – 232)  [2.0 %] | 6.3 (2.4 – 10.6)  [1.3 %] | 254 (139 - 395)  [20.8 %] | 2.9 (1.7 – 4.4)  [28.2 %] | 43.4 %  [33.6 %] | 18.7 %  [17.4 %] | 1752 (935 - 4205)  [21.5 %] | -  [100 %] | 20.7 % [25.5 %] | -  [100 %] | 98.0 % | 82.6 % |
| Oh et al., 2021 [23] | 4790 (1150 - 17474)  [0 %] | 38.5 (38.2 – 39.0)  [0 %] | 67.3 %  [0 %] | 27.7 %  [0 %] | 8.9 (7.9 – 10.2)  [0 %] | 61 (27 – 117)  [0 %] | 2.6 (1.3 – 6.0)  [0 %] | 160 (115 – 274)  [0 %] | 2.6 (1.6 – 4.0)  [0 %] | 58.4 %  [0 %] | 57.4 %  [0 %] | 1873 (1006 - 6587)  [57.4 %] | 100 (49 - 297)  [0 %] | 32.6 % [57.4 %] | 0 %  [0 %] | 100 % | 100 % |
| Yao H et al., 2021 [24] | 3094 (1981 - 15616)  [0 %] | 39.2 (38.9 – 39.8)  [0 %] | 53.6 %  [0 %] | 12.5 %  [0 %] | 10.0 (8.9 – 11.5)  [0 %] | 288 (137 - 372)  [0 %] | 13.4 (6.5 – 18.6)  [0 %] | 151 (105 - 242)  [0 %] | 3.7 (2.2 – 4.9)  [0 %] | 25.5 %  [4.2 %] | 54.9 %  [69.6 %] | 1693 (881 - 2637)  [69.6 %] | 65 (38 - 139)  [0 %] | 9.8 % [69.6 %] | 36.3 %  [0 %] | 100 % | 95.8 % |
| Yao S et al., 2021 [25] | 1809 (877 - 6896)  [67.2 %] | 36.8 (36.5 – 39.0)  [0 %] | 43.7 %  [0 %] | 9.2 %  [0 %] | 11.6 (8.9 – 13.5)  [0 %] | 190 (99 - 266)  [0 %] | 5.7 (3.7 – 8.0)  [0 %] | 120 (82 - 194)  [0.9 %] | 2.9 (2.2 – 4.0)  [0.6 %] | 23.1 %  [0.3 %] | 48.1 %  [70.1 %] | 2650 (1165 - 4972)  [69.3 %] | 22 (16 - 40)  [0 %] | 34.6 % [69.3 %] | 75.3 %  [0 %] | 100 % | 32.8 % |
| Ammouri et al., 2022 [26] | 105 (83 - 119)  [0 %] | 39.0 (37.0 – 39.0)  [0 %] | 16.3 %  [0 %] | 6.7 %  [0 %] | 11.0 (9.0 – 12.0)  [0 %] | 200 (168 - 230)  [0 %] | 6.7 (5.8 – 7.2)  [0 %] | 126 (100 - 200)  [0 %] | 2.0 (1.4 – 2.6)  [0 %] | 93.8 %  [92.3 %] | -  [100 %] | -  [100 %] | 69 (32 - 198)  [90.4 %] | -  [100 %] | 29.8 %  [0 %] | 100 % | 7.7 % |
| He et al., 2022 [27] | 304 (105 - 1345)  [5.0 %] | 39.2 (38.7 – 40.0)  [3.3 %] | 74.4 %  [0 %] | 47.9 %  [0 %] | 10.8 (9.5 – 12.6)  [0.8 %] | 155 (80 - 237)  [0 %] | 4.3 (1.9 – 7.0)  [0 %] | 178 (114 - 255)  [3.3 %] | 2.2 (1.2 – 2.7)  [0 %] | 21.5 %  [0 %] | 42.1 %  [5.8 %] | 463 (183 - 1614)  [5.8 %] | 51 (23 - 114)  [0 %] | 9.9 % [8.3 %] | 70.2 %  [0 %] | 99.2 % | 96.7 % |
| Bilston et al., 2022 [12]* | 1610 (903 - 3386)  [0.1 %] | 37.3 (37.3 – 38.3)  [0 %] | 32.6 %  [0 %] | 11.7 %  [0 %] | 9.6 (8.1 – 11.6)  [0.2 %] | 137 (62 - 249)  [0.2 %] | 6.8 (3.5 – 12.3)  [0.2 %] | 167 (111 - 273)  [48.8 %] | 3.2 (2.0 – 4.7)  [37.3 %] | 11.1 %  [0 %] | 8.3 %  [97.3 %] | 4706 (2006 - 9178)  [95.8 %] | 68 (31 - 179)  [19.7 %] | 57.9 % [95.8 %] | 18.1 %  [0 %] | 99.9 % | 75.0 % |

Descriptive statistics are shown as median ± quartiles, or count with percentage, respectively. Missing data rate in parentheses. *All sIL-2R data were received in U/mL. ASAT, aspartate aminotransferase. NK, natural killer. OHI, optimized HLH inflammatory. sIL-2R, soluble interleukin-2 receptor.

**Supplement Table 10. Validation of original, modified and our newly developed HLH diagnostic criteria sets and biomarkers (with quality criteria).**

|  | Fulfilled criteria or cut-off | Fardet et al., 2016 [9] | Debaugnies et al., 2016 [10] | Horrillo et al., 2019 [20] | Meena et al., 2020 [14] | Debaugnies et al., 2021 [19] | Lopez Marcos et al., 2021 [21] | Smits et al., 2021 [22] | Oh et al., 2021 [23] | Yao H et al., 2021 [24] | Yao S et al., 2021 [25] | Ammouri et al., 2022 [26] | He et al., 2022 [27] | Bilston et al., 2022 [12] | Youden’s index mean | Youden’s index mean (ICU cohorts)**** | Validation cohorts performing worse than respective HLH-2004 (cut-off 4) [n, p-value]***** |
| --- | --- | --- | --- | --- | --- | --- | --- | --- | --- | --- | --- | --- | --- | --- | --- | --- | --- |
| HLH-2004 [4] | 4* | 90.6, 84.6, 85.8, 82.7, 88.5, 78.9, 0.685 | 89.7, 81.3, 77.1, 82.7, 60.0, 91.5, 0.598 | 93.4, 86.5, 97.6, 79.7, 74.5, 98.2, 0.773 | 97.6, 93.9, 90.0, 94.0, 25.7, 99.8, 0.840 | 94.3, 92.5, 64.3, 96.2, 69.2, 95.3, 0.605 | 81.9, 70.1, 78.4, 66.7, 49.2, 88.2, 0.450 | 94.9, 89.3, 92.9, 86.1, 85.5, 93.2, 0.789 | 83.3, 75.2, 90.0, 53.7, 74.0, 78.6, 0.437 | 98.9, 95.2, 98.2, 93.8, 88.7, 99.1, 0.920 | 99.9, 98.3, 95.2, 99.6, 99.0, 98.0, 0.948 | 100, 100, 100, 100, 100, 100, 1.000 | 89.6, 79.3, 71.2, 91.7, 92.9, 67.7, 0.629 | 94.6, 91.0, 83.7, 91.9, 55.8, 97.9, 0.755 | 0.725 | 0.723 | - |
|  | 5 | 90.6, 67.7, 48.1, 98.1, 97.5, 54.8, 0.462 | 89.7, 86.3, 54.3, 97.1, 86.4, 86.3, 0.514 | 93.4, 82.0, 57.1, 97.1, 92.3, 78.8, 0.542 | 97.6, 98.0, 60.0, 98.9, 54.5, 99.1, 0.588 | 94.3, 93.3, 50.0, 99.1, 87.5, 93.8, 0.491 | 81.9, 81.9, 48.6, 95.6, 81.8, 81.9, 0.442 | 94.9, 87.2, 75.7, 97.5, 96.4, 81.9, 0.732 | 83.3, 79.2, 78.3, 80.5, 85.5, 71.7, 0.588 | 98.9, 92.3, 76.8, 100, 100, 89.6, 0.768 | 99.9, 94.5, 81.7, 100, 100, 92.8, 0.817 | 100, 98.6, 85.0, 100, 100, 98.4, 0.850 | 89.6, 62.0, 37.0, 100, 100, 51.1, 0.370 | 94.6, 94.2, 59.2, 98.5, 82.9, 95.2, 0.577 | 0.595 | 0.540 | 12, 0.003 |
| aHLH-2004 | 4 | 91.2, 82.7, 77.8, 90.4, 92.6, 72.3, 0.682 | 87.5, 82.7, 65.7, 88.5, 65.7, 88.5, 0.542 | 85.7, 81.1, 73.8, 85.5, 75.6, 84.3, 0.593 | 96.6, 96.4, 80,0, 96.8, 36.4, 99.5, 0.768 | 95.6, 93.3, 64.3, 97.2, 75.0, 95.4, 0.615 | 80.1, 75.6, 70.3, 77.8, 56.5, 86.4, 0.480 | 93.2, 87.2, 84.3, 89.9, 88.1, 86.6, 0.742 | 85.9, 80.2, 88.3, 68.3, 80.3, 80.0, 0.566 | 98.8, 95.8, 98.2, 94.6, 90.2, 99.1, 0.929 | 99.8, 96.6, 88.5, 100, 100, 95.3, 0.885 | 100, 99.5, 95.0, 100, 100, 99.5, 0.950 | 84.6, 69.4, 53.4, 93.8, 92.9, 57.0, 0.472 | 96.4, 93.8, 80.6, 95.4, 68.1, 97.6, 0.760 | 0.691 | 0.692 | 8, 0.581 |
| oHLH-2004 | 4 | 92.3, 76.7, 63.6, 97.1, 97.2, 63.1, 0.607 | 87.4, 83.5, 65.7, 89.4, 67.6, 88.6, 0.551 | 82.4, 74.8, 47.6, 91.3, 76.9, 74.1, 0.389 | 97.7, 97.8, 70.0, 98.4, 50.0, 99.3, 0.684 | 95.5, 92.5, 57.1, 97.2, 72.7, 94.5, 0.543 | 77.2, 77.2, 51.4, 87.8, 63.3, 81.4, 0.391 | 91.5, 85.2, 80.0, 89.9, 87.5, 83.5, 0.699 | 86.0, 83.2, 85.0, 80.5, 86.4, 78.6, 0.655 | 98.6, 95.2, 89.3, 98.2, 96.2, 94.8, 0.875 | 99.7, 95.4, 84.6, 100, 100, 93.8, 0.846 | 96.4, 97.1, 70.0. 100, 100, 96.9, 0.700 | 83.7, 66.1, 47.9, 93.8, 92.1, 54.2, 0.417 | 96.7, 94.2, 73.5, 96.7, 73.5, 96.7, 0.702 | 0.620 | 0.614 | 12, 0.003 |
| revHLH-2004 | 3 | 90.6, 80.1, 96.3, 54.8, 76.8, 90.5, 0.511 | 89.7, 69.8, 94.3, 61.5, 45.2, 97.0, 0.558 | 93.4, 49.5, 100, 18.8, 42.9, 100, 0.188 | 97.6, 83.6, 100, 83.2, 12.0, 100, 0.832 | 94.3, 90.0, 92.9, 89.6, 54.2, 99.0, 0.825 | 81.9, 50.4, 97.3, 31.1, 36.7, 96.6, 0.284 | 95.1, 77.2, 98.6, 58.2, 67.6, 97.9, 0.568 | 81.8, 70.3, 93.3, 36.6, 68.3, 78.9, 0.299 | 95.5, 68.5, 94.6, 55.4, 51.5, 95.4, 0.500 | 99.5, 97.4, 97.1, 97.5, 94.4, 98.8, 0.947 | 100, 98.1, 100, 97.9, 83.3, 100, 0.979 | 89.5, 81.0, 79.5, 83.3, 87.9, 72.7, 0.628 | 94.5, 77.1, 94.9, 75.0, 31.7, 99.2, 0.699 | 0.601 | 0.829 | 12, 0.003 |
|  | 4 | 90.6, 84.6, 85.8, 82.7, 88.5, 78.9, 0.685 | 89.7, 81.3, 77.1, 82.7, 60.0, 91.5, 0.598 | 93.4, 86.5, 97.6, 79.7, 74.5, 98.2, 0.773 | 97.6, 93.9, 90.0, 94.0, 25.7, 99.8, 0.840 | 94.3, 92.5, 64.3, 96.2, 69.2, 95.3, 0.605 | 81.9, 70.1, 78.4, 66.7, 49.2, 88.2, 0.450 | 95.1, 88.6, 90.0, 87.3, 86.3, 90.8, 0.773 | 81.8, 78.2, 83.3, 70.7, 80.6, 74.4, 0.541 | 95.5, 92.3, 89.3, 93.8, 87.7, 94.6, 0.830 | 99.5, 97.4, 92.3, 99.6, 99.0, 96.8, 0.919 | 100, 100, 100, 100, 100, 100, 1.000 | 89.5, 72.7, 57.5, 95.8, 95.5, 59.7, 0.534 | 94.5, 91.0, 83.7, 91.9, 55.8, 97.9, 0.755 | 0.716 | 0.723 | 4, 0.375 |
|  | 5 | 90.6, 67.7, 48.1, 98.1, 97.5, 54.8, 0.462 | 89.7, 86.3, 54.3, 97.1, 86.4, 86.3, 0.514 | 93.4, 82.0, 57.1, 97.1, 92.3, 78.8, 0.542 | 97.6, 98.0, 60.0, 98.9, 54.5, 99.1, 0.589 | 94.3, 93.3, 50.0, 99.1, 87.5, 93.8, 0.491 | 81.9, 81.9, 48.6, 95.6, 81.8, 81.9, 0.442 | 95.1, 85.2, 71.4, 97.5, 96.2, 79.4, 0.689 | 81.8, 71.3, 60.0, 87.8, 87.7, 60.0, 0.478 | 95.5, 86.9, 60.7, 100, 100, 83.6, 0.607 | 99.5, 91.7, 72.1, 100, 100, 89.4, 0.721 | 100, 98.6, 85.0, 100, 100, 98.4, 0.850 | 89.5, 56.2, 27.4, 100, 100, 47.5, 0.274 | 94.5, 94.2, 59.2, 98.5, 82.9, 95.2, 0.577 | 0.557 | 0.540 | 12, 0.003 |
| shHLH-2004 | 3 | 87.3, 80.1, 90.1, 64.4, 79.8, 80.7. 0.545 | 89.0, 76.3, 80.0, 75.0, 51.9, 91.8, 0.550 | 75.6, 56.8, 97.6, 31.9, 46.6, 95.7, 0.295 | 94.7, 84.9, 90.0, 84.8, 12.0, 99.7, 0.748 | 91.3, 86.7, 64.3, 89.6, 45.0, 95.0, 0.539 | 77.6, 49.6, 94.6, 31.1, 36.1, 93.3, 0.257 | 91.9, 76.5, 95.7, 59.5, 67.7, 94.0, 0.552 | 71.4, 66.3, 85.0, 39.0, 67.1, 64.0, 0.240 | 90.6, 95.2, 98.2, 93.8, 88.7, 99.1, 0.920 | 99.4, 97.7, 96.2, 98.4, 96.2, 98.4, 0.945 | 100, 98.1, 100, 97.9, 83.3, 100, 0.979 | 90.6, 83.5, 79.5, 89.6, 92.1, 74.1, 0.690 | 89.6, 77.4, 89.8, 75.8, 31.3, 98.4, 0.656 | 0.609 | 0.644 | 11, 0.022 |
|  | 4 | 87.3, 73.7, 61.1, 93.3, 93.4, 60.6, 0.544 | 89.0, 84.9, 65.7, 91.3, 71.9, 88.8, 0.571 | 75.6, 72.1, 57.1, 81.2, 64.9, 75.7, 0.383 | 94.7, 95.7, 70.0, 96.3, 30.4, 99.3, 0.663 | 91.3, 92.5, 64.3, 96.2, 69.2, 95.3, 0.605 | 77.6, 68.5, 73.0, 66.7, 47.4, 85.7, 0.396 | 91.9, 85.9, 84.3, 87.3, 85.5, 86.3, 0.716 | 71.4, 67.3, 65.0, 70.7, 76.5, 58.0, 0.357 | 90.6, 88.7, 75.0, 95.5, 89.4, 88.4, 0.705 | 99.4, 94.0, 80.8, 99.6, 98.8, 92.4, 0.804 | 100, 100, 100, 100, 100, 100, 1.000 | 90.6, 65.3, 43.8, 97.9, 97.0, 53.4, 0.417 | 89.6, 89.7, 64.3, 92.9, 52.5, 95.5, 0.572 | 0.595 | 0.634 | 11, 0.022 |
|  | 5 | 87.3, 54.1, 24.7, 100, 100, 46.0, 0.247 | 89.0, 84.2, 40.0, 99.0, 93.3, 83.1, 0.390 | 75.6, 65.8, 14.3, 97.1, 75.0, 65.0, 0.114 | 94.7, 98.0, 20.0, 99.8, 66.7, 98.2, 0.198 | 91.3, 94.2, 50.0, 100, 100, 93.8, 0.500 | 77.6, 79.5, 37.8, 96.7, 82.4, 79.1, 0.345 | 91.9, 76.5, 52.9, 97.5, 94.9, 70.0, 0.503 | 71.4, 51.5, 21.7, 95.1, 86.7, 45.3, 0.168 | 90.6, 79.8, 39.3, 100, 100, 76.7, 0.393 | 99.4, 84.8, 49.0, 100, 100, 82.2, 0.490 | 100, 94.7, 45.0, 100, 100, 94.5, 0.450 | 90.6, 52.1, 20.5, 100, 100, 45.3, 0.205 | 89.6, 90.4, 21.4, 98.9, 70.0, 91.1, 0.203 | 0.324 | 0.349 | 13, < 0.001 |
| HScore [9] | 168* | 96.9, 92.1, 96.3, 85.6, 91.2, 93.7, 0.819 | 92.7, 84.2, 91.4, 81.7, 62.7, 96.6, 0.732 | 83.1, 69.4, 88.1, 58.0, 56.1, 88.9, 0.461 | 95.6, 93.7, 80.0, 94.0, 23.5, 99.5, 0.740 | 97.3, 95.0, 92.9, 95.3, 72.2, 99.0, 0.881 | 82.2, 72.4, 75.7, 71.1, 51.9, 87.7, 0.468 | 91.2, 77.9, 55.7, 97.5, 95.1, 71.3, 0.532 | 78.5, 72.3, 73.3, 70.7, 78.6, 64.4, 0.441 | 99.0, 89.9, 98.2, 85.7, 77.5, 99.0, 0.839 | 98.8, 93.1, 78.8, 99.2, 97.6, 91.7, 0.780 | 99.8, 99.0, 95.0, 99.5, 95.0, 99.5, 0.945 | 90.9, 77.7, 67.1, 93.8, 94.2, 65.2, 0.609 | 97.1, 93.3, 88.8, 93.9, 64.0, 98.6, 0.826 | 0.698 | 0.811 | 7, 1.000 |
|  | 169** | 96.9, 92.1, 96.3, 85.6, 91.2, 93.7, 0.819 | 92.7, 83.5, 88.6, 81.7, 62.0, 95.5, 0.703 | 83.1, 70.3, 88.1, 59.4, 56.9, 89.1, 0.475 | 95.6, 93.7, 80.0, 94.0, 23.5, 99.5, 0.740 | 97.3, 95.0, 92.9, 95.3, 72.2, 99.0, 0.881 | 82.2, 78.7, 73.0, 81.1, 61.4, 88.0, 0.541 | 91.2, 77.9, 55.7, 97.5, 95.1, 71.3, 0.532 | 78.5, 72.3, 73.3, 70.7, 78.6, 64.4, 0.441 | 99.0, 90.5, 98.2, 86.6, 78.6, 99.0, 0.848 | 98.8, 92.2, 76.0, 99.2, 97.5, 90.6, 0.751 | 99.8, 99.5, 95.0, 100, 100, 99.5, 0.950 | 90.9, 76.9, 65.8, 93.8, 94.1, 64.3, 0.595 | 97.1, 93.5, 88.8, 94.1, 64.9, 98.6, 0.829 | 0.700 | 0.811 | 7, 1.000 |
|  | optimal*** | 177; 96.9, 91.4, 92.0, 90.4, 93.7, 87.9, 0.824 | 168; 92.7, 84.2, 91.4, 81.7, 62.7, 96.6, 0.732 | 177; 83.1, 74.8, 85.7, 68.1, 62.1, 88.7, 0.538 | 167; 95.6, 93.7, 90.0, 93.8, 25.0, 99.8, 0.838 | 120; 97.3, 90.8, 100, 89.6, 56.0, 100, 0.896 | 171; 82.2, 78.7, 73.0, 81.1, 61.4, 88.0, 0.541 | 123;  91.2, 81.9, 87.1, 77.2, 77.2, 87.1, 0.644 | 164; 78.5, 74.3, 78.3, 68.3, 78.3, 68.3, 0.466 | 173; 99.0, 94.6, 98.2, 92.9, 87.3, 99.0, 0.911 | 106; 98.8, 93.1, 98.1, 91.0, 82.3, 99.1, 0.891 | 135; 99.8, 96.6, 100, 96.3, 74.1, 100, 0.963 | 151; 90.9, 85.1, 83.6, 87.5, 91.0, 77.8, 0.711 | 157; 97.1, 92.3, 94.9, 92.0, 59.2, 99.3, 0.869 | - | - | - |
| Iteration 1 | 5 | 92.3, 69.2, 50.0, 99.0, 98.8, 56.0, 0.490 | 90.3, 87.1, 68.6, 93.3, 77.4, 89.8, 0.618 | 78.7, 66.7, 19.0, 95.7, 72.7, 66.0, 0.147 | 97.4, 98.0, 70.0, 98.6, 53.8, 99.3, 0.686 | 96.4, 91.7, 57.1, 96.2, 66.7, 94.4, 0.534 | 77.9, 77.2, 48.6, 88.9, 64.3, 80.8, 0.375 | 90.8, 85.2, 78.6, 91.1, 88.7, 82.8, 0.697 | 84.0, 79.2, 76.7, 82.9, 86.8, 70.8, 0.596 | 98.8, 91.1, 75.0, 99.1, 97.7, 88.8, 0.741 | 99.5, 90.2, 67.3, 100, 100, 87.8, 0.673 | 98.2, 95.7, 55.0, 100, 100, 95.4, 0.550 | 84.3, 58.7, 31.5, 100, 100, 49.0, 0.315 | 94.7, 58.2, 93.2,  97.5, 74.0, 95.0, 0.557 | 0.537 | 0.610 | 11, 0.022 |
|  | 6 | 92.5, 72.2, 54.9, 99.0, 98.9, 58.5, 0.540 | 89.1, 84.9, 65.7, 91.3, 71.9, 88.8, 0.571 | 75.8, 68.5, 31.0, 91.3, 68.4, 68.5, 0.223 | 96.8, 98.0, 60.0, 98.9, 54.5, 99.1, 0.589 | 96.5, 91.7, 50.0, 97.2, 70.0, 93.6, 0.472 | 77.6, 78.0, 54.1, 87.8, 64.5, 82.3, 0.418 | 91.2, 83.2, 71.4, 93.7, 90.9, 78.7, 0.651 | 83.9, 82.2, 83.3, 80.5, 86.2, 76.7, 0.638 | 99.5, 89.9, 69.6, 100, 100, 86.8, 0.696 | 99.6, 89.1, 63.5, 100, 100, 86.5, 0.635 | 98.3, 95.7, 55.0, 100, 100, 95.4, 0.550 | 86.0, 62.0, 37.0, 100, 100, 51.1, 0.370 | 94.7, 94.0, 61.2, 98.0, 78.9, 95.4, 0.592 | 0.534 | 0.437 | 12, 0.003 |
| Iteration 2 | 4 | 87.9, 55.3, 26.5, 100, 100, 46.6, 0.265 | 88.3, 86.3, 60.0, 95.2, 80.8, 87.6, 0.552 | 61.7, 61.3, 14.3, 89.9, 46.2, 63.3, 0.041 | 95.0, 97.8, 50.0, 98.9, 50.0, 98.9, 0.489 | 93.5, 94.2, 57.1, 99.1, 88.9, 94.6, 0.562 | 74.8, 78.7, 56.8, 87.8, 65.6, 83.2, 0.445 | 86.7, 75.2, 54.3, 93.7, 88.4, 69.8, 0.480 | 82.2, 73.3, 60.0, 92.7, 92.3, 61.3, 0.527 | 91.3, 60.7, 64.3, 96.4, 90.0, 84.4, 0.607 | 97.8, 85.6, 52.9, 99.6, 98.2, 83.2, 0.525 | 92.8, 94.2, 40.0, 100, 100, 94.0, 0.400 | 82.7, 56.2, 28.8, 97.9, 95.5, 47.5, 0.267 | 94.4, 92.6, 52.0, 97.6, 72.9, 94.3, 0.497 | 0.435 | 0.526 | 12, 0.003 |
| Iteration 5 | 6 | 90.4, 65.8, 44.4, 99.0, 98.6, 53.4, 0.435 | 89.5, 82.7, 60.0, 90.4, 67.7, 87.0, 0.504 | 67.7, 65.8, 31.0, 87.0, 59.1, 67.4, 0.179 | 94.3, 97.3, 30.0, 98.9, 37.5, 98.4, 0.289 | 96.2, 92.5, 57.1, 97.2, 72.7, 94.5, 0.543 | 78.8, 65.4, 67.6, 64.4, 43.9, 82.9, 0.320 | 89.3, 75.2, 55.7, 92.4, 86.7, 70.2, 0.481 | 72.8, 57.4, 41.7, 80.5, 75.8, 48.5, 0.222 | 97.7, 82.1, 46.4, 100, 100, 78.9, 0.464 | 96.4, 81.6, 40.4, 99.2, 95.5, 79.6, 0.396 | 99.2, 97.1, 70.0, 100, 100, 96.9, 0.700 | 85.4, 51.2, 19.2, 100, 100, 44.9, 0.192 | 92.5, 91.9, 50.0, 97.0, 67.1, 94.1, 0.470 | 0.400 | 0.416 | 13, < 0.001 |
| Iteration 6 | 6 | 91.6, 66.5, 45.1, 100, 100, 53.9, 0.451 | 89.1, 82.7, 57.1, 91.3, 69.0, 86.4, 0.485 | 64.1, 62.2, 28.6, 82.6, 50.0, 65.5, 0.112 | 94.2, 97.1, 40.0, 98.4, 36.4, 98.6, 0.384 | 95.1, 93.3, 57.1, 98.1, 80.0, 94.5, 0.553 | 78.0, 72.4, 67.6, 74.4, 52.1, 84.8, 0.420 | 89.6, 71.8, 45.7, 94.9, 88.9, 66.4, 0.407 | 71.0, 54.5, 33.3, 85.4, 76.9, 46.7, 0.187 | 96.6, 79.8, 39.3, 100, 100, 76.7, 0.393 | 97.8, 80.7, 35.6, 100, 100, 78.5, 0.356 | 99.8, 96.2, 60.0, 100, 100, 95.9, 0.600 | 85.7, 48.8, 15.1, 100, 100, 43.6, 0.151 | 91.7, 91.2, 45.9, 96.7, 63.4, 93.6, 0.427 | 0.379 | 0.469 | 13, < 0.001 |
| Iteration 8 | 6 | 89.2, 74.1, 63.6, 90.4, 91.2, 61.4, 0.540 | 89.8, 84.9, 68.6, 90.4, 70.6, 89.5, 0.590 | 63.1, 60.4, 66.7, 16.7, 47.7, 68.7, 0.167 | 95.5, 97.5, 60.0, 98.4, 46.2, 99.1, 0.584 | 95.7, 92.5, 57.1, 97.2, 72.7, 94.5, 0.543 | 76.8, 64.6, 86.5, 55.6, 44.4, 90.9, 0.420 | 87.5, 75.8, 55.7, 93.7, 88.6, 70.5, 0.494 | 72.7, 62.4, 55.0, 73.2, 75.0, 52.6, 0.282 | 98.2, 85.1, 55.4, 100, 100, 81.8, 0.554 | 96.5, 82.8, 42.3, 100, 100, 80.3, 0.423 | 97.0, 96.2, 65.0, 99.5, 92.9, 96.4, 0.645 | 85.6, 68.6, 52.1, 93.8, 92.7, 56.3, 0.458 | 92.7, 91.9, 57.1, 96.1, 64.4, 94.8, 0.533 | 0.479 | 0.564 | 13, < 0.001 |
| Iteration 11 | 5 | 83.6, 58.6, 33.3, 98.1, 96.4, 48.6, 0.314 | 86.0, 84.9, 65.7, 91.3, 71.9, 88.8, 0.571 | 61.1, 63.1, 31.0, 82.6, 52.0, 66.3, 0.136 | 95.4, 98.0, 40.0, 99.3, 57.1, 98.6, 0.393 | 93.4, 92.5, 64.3, 96.2, 69.2, 95.3, 0.605 | 76.4, 72.4, 32.4, 88.9, 54.5, 76.2, 0.213 | 88.2, 71.8, 44.3, 96.2, 91.2, 66.1, 0.405 | 77.7, 68.3, 55.0, 87.8, 86.8, 57.1, 0.428 | 97.3, 85.7, 57.1, 100, 100, 82.4, 0.571 | 97.6, 87.4, 59.6, 99.2, 96.9, 85.2, 0.588 | 98.4, 93.8, 35.0, 100, 100, 93.5, 0.350 | 86.8, 66.9, 46.6, 97.9, 97.1, 54.7, 0.445 | 86.2, 90.2, 19.4, 98.9, 67.9, 90.9, 0.183 | 0.400 | 0.499 | 12, < 0.001 |
| Iteration 12 | 5 | 85.6, 50.8, 19.1, 100, 100, 44.3, 0.191 | 90.1, 87.1, 54.3, 98.1, 90.5, 86.4, 0.524 | 60.8, 62.2,  9.5, 94.2, 50.0, 63.1, 0.037 | 96.5, 97.5, 30.0, 99.1, 42.9, 98.4, 0.291 | 96.6, 94.2, 57.1, 99.1, 88.9, 94.6, 0.562 | 76.5, 76.4, 48.6, 87.8, 62.1, 80.6, 0.364 | 86.6, 74.5, 54.3, 92.4, 86.4, 69.5, 0.467 | 77.5, 56.4, 31.7, 92.7, 86.4, 48.1, 0.243 | 94.2, 80.4, 41.1, 80.4, 100, 77.2, 0.411 | 97.4, 83.9, 47.1, 99.6, 98.0, 81.5, 0.467 | 92.6, 93.8, 35.0, 100, 100, 93.5, 0.350 | 82.0, 49.6, 16.4, 100, 100, 44.0, 0.164 | 91.8, 91.3, 32.7, 98.5, 72.7, 92.3, 0.312 | 0.337 | 0.427 | 13, < 0.001 |
|  | 6 | 90.4, 58.3, 31.5, 100, 100, 48.4, 0.315 | 88.5, 83.5, 51.4, 94.2, 75.0, 85.2, 0.457 | 70.3, 67.6, 28.6, 91.3, 66.7, 67.7, 0.199 | 94.4, 98.0, 30.0, 99.5, 60.0, 98.4, 0.295 | 96.6, 93.3, 57.1, 98.1, 80.0, 94.5, 0.553 | 78.5, 69.3, 67.6, 70.0, 48.1, 84.0, 0.376 | 90.0, 81.9, 72.9, 89.9, 86.4, 78.9, 0.627 | 75.3, 61.4, 45.0, 85.4, 81.8, 51.5, 0.304 | 98.2, 85.7, 57.1, 100, 100, 82.4, 0.571 | 97.9, 84.8, 50.0, 99.6, 98.1, 82.4, 0.496 | 98.9, 96.6, 65.0, 100, 100, 96.4, 0.650 | 87.6, 57.9, 30.1, 100, 100, 48.5, 0.301 | 94.1, 92.8, 52.0, 97.7, 73.9, 94.3, 0.498 | 0.434 | 0.424 | 13, < 0.001 |
| Iteration 13 | 5 | 86.7, 53.8, 24.7, 99.0, 97.6, 45.8, 0.237 | 87.4, 87.8, 62.9, 96.2, 84.6, 88.5, 0.590 | 66.4, 66.7, 40.5, 82.6, 58.6, 69.5, 0.231 | 96.2, 97.5, 50.0, 98.6, 45.5, 98.8, 0.486 | 95.2, 91.7, 50.0, 97.2, 70.0, 93.6, 0.472 | 74.2, 74.8, 29.7, 93.3, 64.7, 76.4, 0.231 | 86.9, 75.8, 54.3, 94.9, 90.5, 70.1, 0.492 | 76.7, 59.4, 35.0, 95.1, 91.3, 50.0, 0.301 | 93.0, 76.8, 30.4, 100, 100, 74.2, 0.304 | 98.6, 83.0, 43.3, 100, 100, 80.5, 0.433 | 97.8, 92.8, 25.0, 100, 100, 92.6, 0.250 | 87.3, 49.6, 16.4, 100, 100, 44.0, 0.164 | 91.3, 90.7, 29.6, 98.2, 67.4, 91.9, 0.278 | 0.344 | 0.479 | 13, < 0.001 |
|  | 6 | 91.9, 59.0, 32.7, 100, 100, 48.8, 0.327 | 87.9, 82.0, 48.6, 93.3, 70.8, 84.3, 0.418 | 65.8, 64.9, 28.6, 87.0, 57.1, 66.7, 0.155 | 94.4, 97.3, 30.0, 98.9, 37.5, 98.4, 0.289 | 95.7, 93.3, 57.1, 98.1, 80.0, 94.5, 0.553 | 78.2, 74.0, 62.2, 78.9, 54.8, 83.5, 0.411 | 90.2, 79.2, 64.3, 92.4, 88.2, 74.5, 0.567 | 73.9, 58.4, 36.7, 90.2, 84.6, 49.3, 0.269 | 97.2, 83.9, 51.8, 100, 100, 80.6, 0.518 | 98.7, 83.9, 46.2, 100, 100, 81.3, 0.462 | 99.6, 95.7, 55.0, 100, 100, 95.4, 0.550 | 88.9, 56.2, 27.4, 100, 100, 47.5, 0.274 | 93.3, 92.8, 51.0, 97.9, 74.6, 94.2, 0.489 | 0.406 | 0.421 | 13, < 0.001 |
|  | 7 | 91.6, 51.1, 19.8, 100, 100, 44.4, 0.198 | 89.0, 84.2, 54.3, 94.2, 76.0, 86.0, 0.485 | 66.8, 63.1, 9.5, 95.7, 57.1, 63.5, 0.052 | 93.1, 97.8, 20.0, 99.5, 50.0, 98.2, 0.195 | 95.7, 93.3, 50.0, 99.1, 87.5, 93.8, 0.491 | 79.9, 78.7, 51.4, 90.0, 67.9, 81.8, 0.414 | 89.5, 71.8, 47.1, 93.7, 86.8, 66.7, 0.408 | 75.5, 57.4, 31.7, 95.1, 90.5, 48.8, 0.268 | 97.1, 77.4, 32.1, 100, 100, 74.7, 0.321 | 98.2, 81.0, 36.5, 100, 100, 78.7, 0.365 | 99.8, 94.7, 45.0, 100, 100, 94.5, 0.450 | 87.3, 48.8, 15.1, 100, 100, 43.6, 0.151 | 93.4, 91.3, 30.6, 98.7, 75.0, 92.1, 0.294 | 0.315 | 0.343 | 13, < 0.001 |
| Ferritin [µg/L] | 9083* | 86.5, 56.8, 34.0, 96.6, 94.5, 45.7, 0.306 | 77.4, 73.7, 42.9, 90.6, 71.4, 74.4, 0.335 | 65.0, 63.8, 23.8, 90.5, 62.5, 64.0, 0.143 | 86.1, 95.1, 50.0, 96.1, 22.7, 98.8, 0.461 | 94.1, 95.8, 71.4, 99.1, 90.9, 96.3, 0.705 | 76.7, 69.0, 10.7, 96.6, 60.0, 69.5, 0.073 | 69.8, 56.7, 27.5, 87.7, 70.4, 53.3, 0.152 | 73.7, 62.4, 48.3, 82.9, 80.6, 52.3, 0.313 | 80.0, 74.4, 64.3, 79.5, 61.0, 81.7, 0.438 | 90.0, 28.9, 21.4, 100, 100, 12.0, 0.214 | 100, 93.3, 30.0, 100, 100, 93.1, 0.300 | 88.4, 42.6, 4.3, 100, 100, 41.1, 0.043 | 90.3, 91.0, 59.8, 94.7, 58.0, 95.1, 0.545 | 0.310 | 0.583 | 12, 0.003 |
|  | optimal*** | 1985; 86.5, 83.4, 86.9, 77.3, 86.9, 77.3, 0.642 | 3353; 77.4, 71.7, 68.6, 73.4, 58.5, 81.0, 0.420 | 1351; 65.0, 61.0, 76.2, 50.8, 50.8, 76.2, 0.270 | 1197; 86.1, 70.6, 90.0, 70.1, 6.5, 99.7, 0.601 | 1799; 94.1, 82.5, 92.9, 81.1, 39.4, 98.9, 0.740 | 802; 76.7, 71.3, 82.1, 66.1, 53.5, 88.6, 0.482 | 1140; 69.8, 69.4, 78.3, 60.0, 67.5, 72.2, 0.383 | 5061; 73.7, 70.3, 65.0, 78.0, 81.3, 60.4, 0.430 | 9574; 80.0, 76.2, 64.3, 82.1, 64.3, 82.1, 0.464 | 481; 90.0, 89.5, 90.3, 81.8, 97.9, 47.4, 0.721 | 692; 100, 100, 100, 100, 100, 100, 1.000 | 202; 88.4, 84.3, 84.1, 84.8, 89.2, 78.0, 0.688 | 6843; 90.3, 89.5, 73.2, 91.5, 51.1, 96.6, 0.647 | - | - | - |
| sIL-2R [U/mL] | 4621* | n.a. | 70.4, 40.6, 5.0, 100, 100, 38.7, 0.050 | n.a. | n.a. | 83.9, 91.7, 28.6, 100, 100, 91.4, 0.286 | 61.1, 45.5, 33.3, 100, 100, 25.0, 0.333 | 81.3, 65.8, 37.9, 93.2, 84.6, 60.4, 0.312 | 72.0, 51.2, 37.5, 90.9, 92.3, 33.3, 0.284 | 89.6, 9.8, 4.2, 100, 100, 6.1, 0.042 | 90.4, 36.4, 32.7, 100, 100, 8.1, 0.327 | n.a. | 75.0, 46.5, 14.3, 97.7, 90.9, 41.7, 0.120 | 58.2, 57.9, 55.6, 63.6, 78.9, 36.8, 0.192 | 0.206 | 0.286 | 9, 0.004 |
|  | optimal*** | n.a. | 462; 70.4, 65.6, 60.0, 75.0, 80.0, 52.9, 0.350 | n.a. | n.a. | 576; 83.9, 84.2, 78.6, 84.9, 40.7, 96.8, 0.635 | 2849; 61.1, 63.6, 55.6, 100, 100, 33.3, 0.556 | 1171; 81.3, 76.1, 91.4, 61.0, 69.7, 87.8, 0.524 | 1829; 72.0, 67.4, 62.5, 81.8, 90.9, 42.9, 0.443 | 847; 89.6, 82.4, 81.3, 100, 100, 25.0, 0.813 | 753; 90.4, 87.9, 88.1, 83.3, 98.9, 29.4, 0.715 | n.a. | 740; 75.0, 71.1, 58.6, 90.9, 91.1, 58.0, 0.495 | 4285; 58.2, 60.5, 59.3, 63.6, 80.0, 38.9, 0.229 | - | - | - |
| OHI index [11] | sIL-2R > 3900 U/mL and ferritin > 1000 µg/L | n.a. | 52.5, 32.1, 5.0, 100, 100, 29.6, 0.050 | n.a. | n.a. | 64.3, 91.7, 28.6, 100, 100, 91.4, 0.286 | 66.7, 45.5, 33.3, 100, 100, 25.0, 0.333 | 66.2, 64.9, 36.2, 96.2, 91.3, 58.0, 0.324 | 65.8, 53.5, 40.6, 90.1, 92.9, 34.5, 0.315 | 55.2, 15.7, 10.4, 100, 100, 6.5, 0.104 | 68.3, 40.2, 36.6, 100, 100, 8.6, 0.366 | n.a. | 58.1, 48.6, 16.2, 100, 100, 43.0, 0.162 | 58.8, 60.5, 63.0, 54.5, 77.3, 37.5, 0.175 | 0.235 | 0.286 | 9, 0.004 |

Results shown as area under the curve [%], accuracy [%], sensitivity [%], specificity [%], positive predictive value [%], negative predictive value [%], Youden’s index. *Optimal cut-off in our previous study [15]. **Optimal cut-off within the developmental dataset [9]. ***Optimal cut-off determined for each study, shown with corresponding accuracy [%], sensitivity [%], specificity [%], positive predictive value [%], negative predictive value [%], and Youden’s index. ****Only Meena et al. [14] and Debaugnies et al. [19] considered. *****Statistical analyses performed using sign test. aHLH-2004, adjusted HLH-2004 criteria. HLH, Hemophagocytic Lymphohistiocytosis. n.a., not applicable. OHI, optimized HLH inflammatory. oHLH-2004, optimized HLH-2004 criteria. revHLH-2004, revised HLH-2004 criteria. shHLH-2004, shortened HLH-2004 criteria. sIL-2R, soluble interleukin-2 receptor.

**Supplement Table 11. Sensitivities of different ferritin cut-offs to diagnose HLH.**

|  | Cut-off | Fardet et al., 2016 [9] | Debaugnies et al., 2016 [10] | Horrillo et al., 2019 [20] | Meena et al., 2020 [14] | Debaugnies et al., 2021 [19] | Lopez Marcos et al., 2021 [21] | Smits et al., 2021 [22] | Oh et al., 2021 [23] | Yao H et al., 2021 [24] | Yao S et al., 2021 [25] | Ammouri et al., 2022 [26] | He et al., 2022 [27] | Bilston et al., 2022 [12] | Mean | Mean (ICU cohorts)** |
| --- | --- | --- | --- | --- | --- | --- | --- | --- | --- | --- | --- | --- | --- | --- | --- | --- |
| Ferritin [µg/L] | Highest cut-off with sensitivity of 100 % | 72 | 501 | 494 | 533 | 794 | 237 | 52 | 217 | 774 | 23 | 692 | 15 | 1018 | 421 | 664 |
|  | Highest cut-off with sensitivity of 99 % | 371 | 501 | 494 | 533 | 794 | 237 | 52 | 217 | 774 | 48 | 692 | 15 | 1468 | 481 | 664 |
|  | Highest cut-off with sensitivity of 98 % | 847 | 501 | 494 | 533 | 794 | 237 | 76 | 233 | 1572 | 249 | 692 | 18 | 1468 | 593 | 664 |
|  | Highest cut-off with sensitivity of 95 % | 1000 | 595 | 562 | 533 | 794 | 273 | 350 | 598 | 1627 | 347 | 1450 | 54 | 1647 | 756 | 664 |
|  | 300 | 99.3 % | 100 % | 100 % | 100 % | 100 % | 96.4 % | 95.7 % | 96.7 % | 100 % | 96.1 % | 100 % | 76.8 % | 100 % | 97.0 % | 100 % |
|  | 400 | 98.7 % | 100 % | 100 % | 100 % | 100 % | 92.9 % | 94.2 % | 95.0 % | 100 % | 91.3 % | 100 % | 68.1 % | 100 % | 95.4 % | 100 % |
|  | 500 | 98.7 % | 100 % | 97.6 % | 100 % | 100 % | 85.7 % | 89.9 % | 95.0 % | 100 % | 89.3 % | 100 % | 65.2 % | 100 % | 94.0 % | 100 % |
|  | 1000 | 95.4 % | 88.6 % | 83.3 % | 90.0 % | 92.9 % | 71.4 % | 78.3 % | 88.3 % | 98.2 % | 77.7 % | 95.0 % | 55.1 % | 100 % | 85.7 % | 91.5 % |
|  | 3000* | 68.6 % | 68.6 % | 38.1 % | 60.0 % | 78.6 % | 35.7 % | 50.7 % | 71.7 % | 75.0 % | 35.9 % | 75.0 % | 11.6 % | 82.5 % | 57.8 % | 69.3 % |

Results shown as ferritin cut-offs [µg/L] or sensitivities [%], respectively. *Optimized cut-off for HLH-2004 criteria within the developmental dataset [9]. **Only Meena et al. [14] and Debaugnies et al. [19] considered.
