# Supplementary figures and images for: Multicenter validation of secondary hemophagocytic lymphohistiocytosis diagnostic criteria
Source: J Intern Med. 2025 Jan 27;297(3):312–27. doi: 10.1111/joim.20065 (PMC11846073; doi:10.1111/joim.20065)

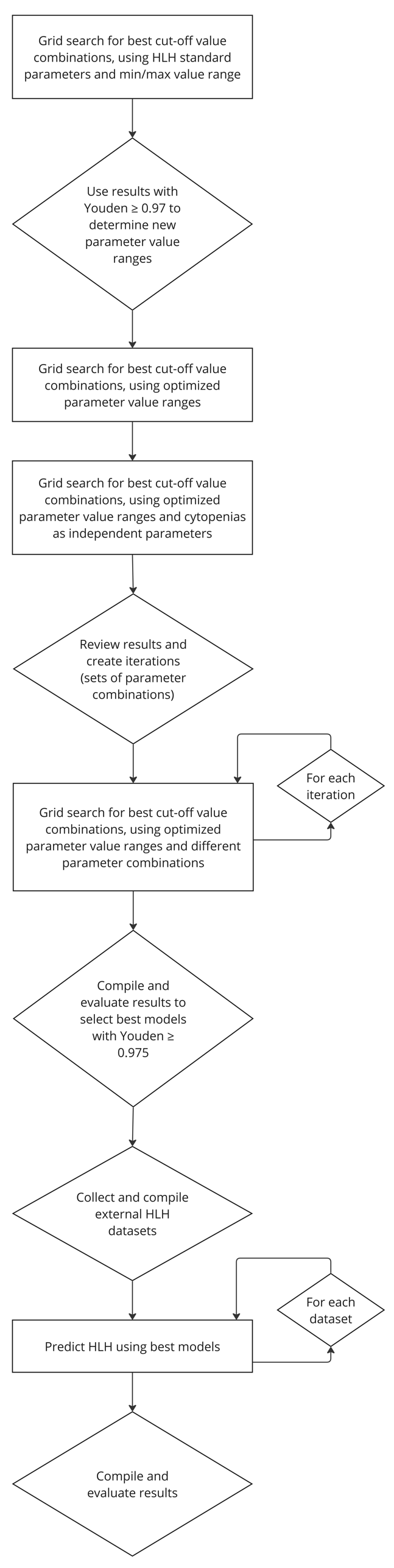

Supplement: Supplementary file 2 — Fig. S1. Optimizing strategy. [file JOIM-297-312-s002.png]
